# Investigating Pb2 CAP-binding domain inhibitors from marine bacteria for targeting the influenza A H5N1

**DOI:** 10.1371/journal.pone.0310836

**Published:** 2025-01-28

**Authors:** Taha A. Kumosani, Aymn T. Abbas, Balogun Basheer, Ahmed M. Hassan, Soonham S. Yaghmoor, Areej H. Alyahiby, Amer H. Asseri, Vivek Dhar Dwivedi, Esam I. Azhar

**Affiliations:** 1 Biochemistry Department, Faculty of Science, King Abdulaziz University, Jeddah, Saudi Arabia; 2 Experimental Biochemistry Unit, King Fahd Medical Research Center, King Abdulaziz University, Jeddah, Saudi Arabia; 3 Special Infectious Agents Unit–BSL3, King Fahd Medical Research Center, King Abdulaziz University, Jeddah, Saudi Arabia; 4 Department of Medical Laboratory Sciences, Faculty of Applied Medical Sciences, King Abdulaziz University, Jeddah, Saudi Arabia; 5 Centre for Artificial Intelligence in Precision Medicines, King Abdul-Aziz University, Jeddah, Saudi Arabia; 6 Center for Global Health Research, Saveetha Institute of Medical and Technical Sciences, Saveetha Medical College and Hospitals, Saveetha University, Chennai, India; 7 Bioinformatics Research Division, Quanta Calculus, Greater Noida, India; American University, ARUBA

## Abstract

The ongoing increase in the prevalence and mutation rate of the influenza virus remains a critical global health issue. A promising strategy for antiviral drug development involves targeting the RNA-dependent RNA polymerase, specifically the PB2-cap binding domain of Influenza A H5N1. This study employs an in-silico approach to inhibit this domain, crucial for viral replication, using potential inhibitors derived from marine bacterial compounds. Utilizing the MTi-OpenScreen web server, we screened a library of compounds to assess their molecular interactions with the target. This process identified four potential inhibitors: CMNPD25830, CMNPD18675, CMNPD18676, and CMNPD27216. Subsequent molecular dynamics simulations, conducted using the Amber software suite, evaluated their binding affinities and dynamic interactions with the PB2 protein. Notably, CMNPD25830 and CMNPD27216 emerged as the most promising candidates, exhibiting higher binding affinities and more favourable interaction profiles compared to the control molecule. Additional analyses, including post-simulation free energy calculations and free energy landscape analysis, strengthened the potential of these compounds as effective PB2-cap binding domain inhibitors. This comprehensive computational investigation identifies CMNPD27216 and CMNPD25830 as standout candidates due to their superior binding energies and dynamic stability, suggesting their strong potential as therapeutic agents against influenza. This research sets the stage for further in vitro validation and optimization of these lead compounds, potentially supporting the development of more effective influenza treatments.

## 1. Introduction

Influenza, a highly contagious viral infection, remains a significant global health concern despite advancements in medical science [1]. The influenza virus’ ability to rapidly mutate and evolve has challenged the development of effective and long-lasting antiviral therapies [2–4]. The PB2 cap-binding domain within the influenza virus polymerase has surfaced as a prime focus for new antiviral drug development [5–7]. As a member of the Orthomyxoviridae family, this virus is enveloped and segmented having a single RNA strand of negative sense [8]. Each segment of their genome forms a structure called viral ribonucleoprotein (vRNA), which is composed of several copies of nuclear proteins. It also includes a viral RNA-dependent RNA polymerase (RdRp) essential for the virus’ life cycle and its ability to infect host cells [9, 10]. The RdRp protein of the virus is a complex structure made up of three different proteins. These proteins are PA, PB1, and PB2 named polymerase acidic protein, polymerase basic protein 1, and polymerase basic protein 2, respectively [6, 9, 11]. These proteins are crucial for the replication and transcribing of the virus’s genetic material within the nucleus of the infected cells. The influenza virus, lacking the ability to form its 5′-terminal 7-methylguanosine (m7G) cap primer necessary for the transcription, employs a process known as “cap-snatching” to attain these 5’ cap primers [6]. In this mechanism, the PB2 cap-binding domain of the viral RdRp seizes the 5′ cap of newly formed host-capped RNAs. Following this, the PA endonuclease domain splits the capped RNA a certain distance (8–14 nucleotides) downstream of the cap structure [12, 13]. The conserved polymerase domain within the PB1 subunit is employed as a primer to facilitate RNA elongation [14]. Since human cells do not possess a similar mechanism to the cap-snatching process, the strategy for developing inhibitors that target PB2 or PA and disrupt this process is considered optimal for the invention of novel anti-influenza therapeutics [14]. VX-787, an inhibitor of PB2, has passed stage II clinical trials and is now enduring evaluation in stage III clinical trials [15]. Though, the evolution of VX-787 has come across some challenges. One plausible explanation is that the pyrimidine-7-azaindole motif of VX-787 can be processed by human aldehyde oxidase (AO), resulting in a suboptimal pharmacokinetic profile [15, 16]. Thus, the exploration of a PB2 inhibitor with a novel chemical structure is a viable strategy for the development of anti-influenza drugs. As numerous recent studies have proven, virtual screening methods are successful in identifying lead compounds with innovative and potential chemical structures in drug discovery [14, 17]. In this report, we present our efforts to identify potent compounds for PB2 inhibitors using *in-silico* drug discovery. This study harnessed an array of bioinformatics tools to discover potential PB2 inhibitors in marine bacteria. We initiated our research with virtual screening to filter and pinpoint potential inhibitors from a database of metabolites derived from marine bacteria. This digital approach facilitates the quick and cost-effective screening of vast compound libraries, effectively shortlisting potential candidates for more detailed analysis.

## 2. Methodology

### 2.1 Target and library preparations

This study employs a computational approach to determine potential inhibitors for the influenza virus’s polymerase PB2 cap-binding domain. The target protein was sourced from the protein data bank (PDB) having PDB ID-4CB5 [18, 19] and the ligands were collected from the marine bacteria database [20], which contains a large collection of compounds suitable for high throughput screening. We prepared the protein using the Dock Prep tool in Chimera [21]. This involved preparing the protein structure by eliminating water molecules and any attached ligands or ions, followed by adding hydrogen atoms to precise the protonation state and optimize the protein’s overall shape.

### 2.2 Virtual screening

We used the MtiOpenScreen web server for the virtual screening, which includes a Lipinski filter to ensure that the screened compounds followed basic pharmacokinetic principles [22–24]. This screening process identified several candidates. We selected four compounds based on their high docking scores, indicating potential high-affinity binding to the target domain.

### 2.3 Re-docking

The selected compounds were then performed with re-docking using the Autodock Vina Chimera plugin [25]. This step validated the initial docking results with a more refined computational model. We docked both the candidate compounds and a reference molecule into the binding site of the target protein. This re-docking process allowed us to compare the binding affinities and shapes of the candidates to a known inhibitor, confirming their potential effectiveness.

### 2.4 Molecular dynamics simulation

The study was initiated with the utilization of MD simulations to examine the dynamic behavior of the influenza polymerase PB2 cap-binding domain. The MD simulations were conducted using the Generalized Amber Force Field (GAFF) as incorporated in the AMBER simulation package (https://ambermd.org/index.php) [26–28]. The AMBER force field is renowned for its versatility in both DNA and proteins, ensuring consistency and reliable simulation results across types of biomolecules [29]. It has parameters for almost all organic molecules, including H, C, N, O, S, P, and halogens [30, 31]. Also, it is well-validated for modelling the structural and dynamic properties of both biomolecules and provides high-quality parameters, making it suitable for this study [32]. The ligand molecules were prepared using the antechamber tool within AMBER, which facilitated the assignment of force field parameters and atomic charges suitable for non-standard residues in the ligands [33]. This preparation is crucial for ensuring accurate interactions within the simulated system. After the ligand preparation, the complex assembly and system configuration were accomplished using the leap tool from AMBER. This process encompassed the integration of each ligand into the target protein’s binding site then immersing each complex in an explicit TIP3P water model, and the addition of appropriate counterions to achieve electro neutrality [28]. The simulation protocol was initiated with a comprehensive energy minimization performed by the SANDER module [34]. This was succeeded by a temperature equilibration phase where each system was heated with time from 0 K to 300 K across a span of 100 picoseconds, utilizing the NVT ensemble (constant number of particles, constant volume and temperature). Following temperature equilibration, the systems were further equilibrated below NPT conditions (constant number of particles constant pressure and temperature) for 1 nanosecond to ensure stable density and thermal conditions, setting the stage for the production of MD simulations. Each complex underwent a production run lasting 100 ns, with the pressure and temperature kept constant at 1 atmosphere and 300 Kelvin, respectively [35]. The Langevin thermostat was employed for temperature regulation, while pressure was managed using the Berendsen barostat [36–38]. Trajectory data harvested from the simulations underwent a rigorous study to examine the stability and conformational dynamics of the complexes. Metrics such as the root-mean-square deviation (RMSD) (usually the backbone atoms), root-mean-square fluctuation (RMSF) of each residue, and the number of persistent hydrogen bonds between each ligand and the protein were computed. These parameters provide insights into the structural fidelity and interaction dynamics across the simulation period.

To further comprehend the collective movements that influence the dynamics of the system, the RG-RMSD-based free energy landscape was further used to determine significant motions from the trajectories. Free energy calculations were also carried out to determine the binding efficiency and favorable conformational states, providing a comprehensive representation of the interaction between the landscape and energetics. Using the AMBER simulation software, this methodology offers a systematic approach to comprehending the dynamic behaviour of the Influenza Polymerase Pb2 CAP-Binding Domain.

### 2.5 Free binding energy

The process is initiated by employing the Amber force field during molecular dynamics simulations [27]. The simulations are carried out on the complex formed by the protein and ligand. The aim is to generate a trajectory that sufficiently explores the various possible conformations and complexes. Following the generation of the trajectory, the Molecular Mechanics/Poisson-Boltzmann Surface Area (MM/PBSA) method is used to compute the free binding energy [39, 40]. This technique combines the energy from molecular mechanics with the energy of solvation, which is further divided into polar and nonpolar components. The energy of polar solvation is calculated using the Poisson-Boltzmann equation, whereas the energy of nonpolar solvation is estimated based on the area accessible to the solvent [41]. The free energy of binding is then obtained by subtracting the free energy of the unbound state from the free energy of the bound state [42].

### 2.6 RG-RMSD-based free energy landscape

The method involves the trajectory generated from molecular dynamics simulations and then assigned into PyMOL for thorough analysis [43]. Utilizing the Geo Measures plugin in PyMOL, the protein-ligand complex’s Root-Mean-Square Deviation (RMSD) and Radius of Gyration (RG) are calculated throughout the trajectory and provide measures of compactness and structural variation, respectively [44, 45]. Plotting these metrics reciprocally generates a two-dimensional free energy landscape that provides a visual representation of the conformational space that the complex traverses during the simulation [46]. We analyze this landscape to identify the most stable conformations and their respective transition paths, providing insights into the complex’s dynamic behaviour and the binding process energetics.

## 3. Result and discussion

### 3.1 Virtual screening

Virtual screening, a powerful computational technique, was utilized in this research to discover bioactive molecules that can bind to specific biological targets. A comprehensive analysis was conducted on 1501 compounds from the Marine Bacteria Database, performed on the MTiopen web server [22]. The selection of compounds was primarily guided by their binding energy values, which were obtained during the screening process. The binding energies of these molecules varied, ranging from -11.3 kcal/mol to -6.7 kcal/mol (as detailed in S1 Table in S1 File). We utilized the 9-n-(3-carboxy-4-hydroxyphenyl)ketomethyl-7-n-methylguanine (93G), a control molecule, which had a binding energy of -7.5 kcal/mol, as a point of reference. Four compounds, i.e., CMNPD25830, CMNPD18675, CMNPD18676, and CMNPD27216, stood out due to their notable binding energies. These compounds, listed in a table with their SMILES and compound IDs, are potential candidates for further investigation and validation in the search for new bioactive molecules (S2 Table in S1 File).

### 3.2 Re-docking and intermolecular analysis

**Fig 1:**
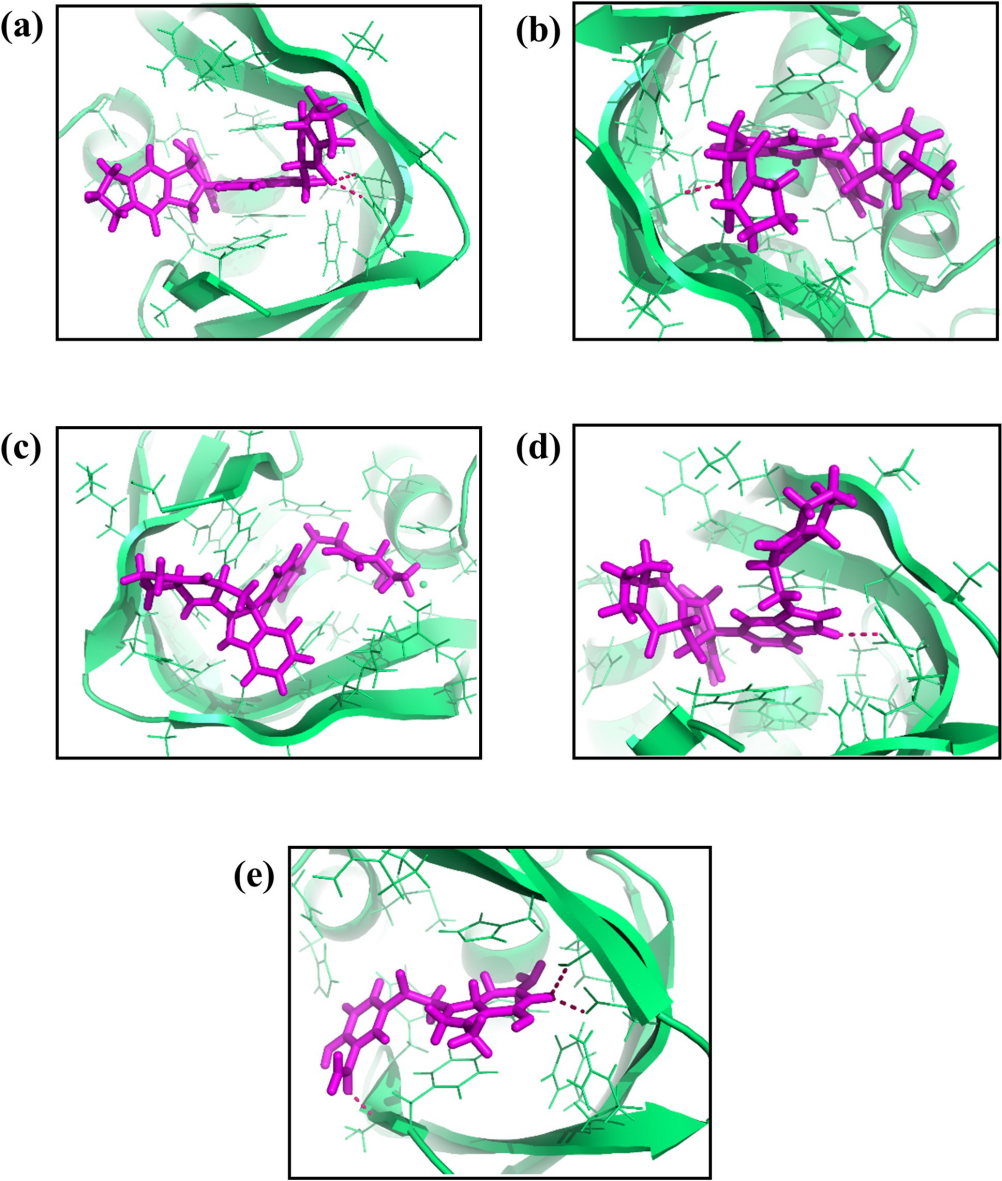
3D interaction diagram of the selected docked complexes with the target influenza A H5N1 and compounds i.e., (a) CMNPD25830 (b) CMNPD18675 (c) CMNPD18676 (d) CMNPD27216 and (e) Control.

**Fig 2:**
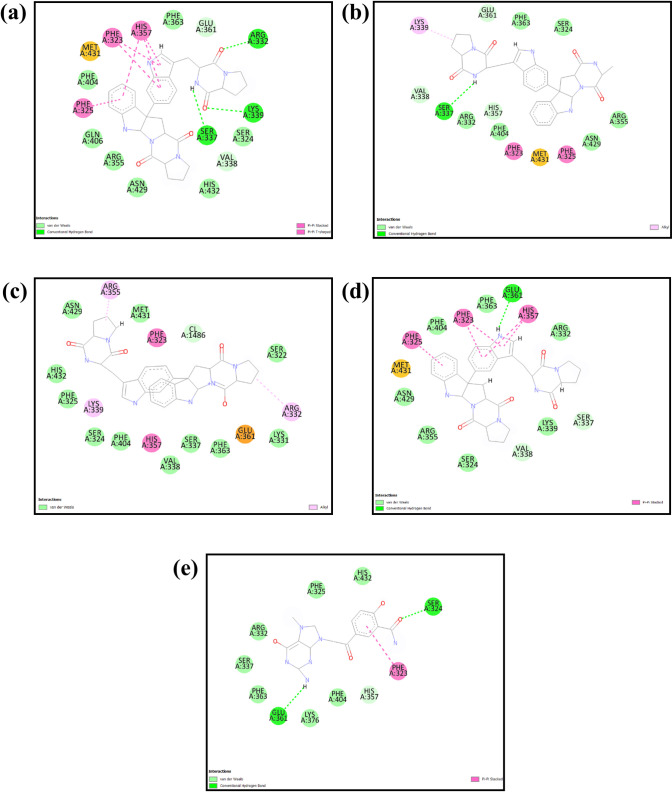
2D interaction diagram of the selected docked complexes with the target influenza A H5N1 and compounds i.e., (a) CMNPD25830 (b) CMNPD18675 (c) CMNPD18676 (d) CMNPD27216 and (e) Control.

We used specific parameters to perform the re-docking procedure in our investigation. The coordinates for the docking grid’s center were determined to be X = -50.0, Y = 4.48, and Z = -3.14. The grid’s dimensions were 20 Å for each of the axes. This screening approach found Four compounds to have remarkably significant binding energies. The binding energies for the compounds CMNPD25830, CMNPD18675, CMNPD18676, and CMNPD27216 were calculated and reported as -11.3, -11.1, -11.3, and -10.9 kcal/mol, respectively. PyMOL software was used to visualize the three-dimensional structures of these compounds [43], including the control, while Biodiscovery Studio produced two-dimensional representations (Figs 1 and 2) [47]. As seen in Figs 1 and 2, this significantly improved our comprehension of their spatial conformations and interactions within the binding site.

The interaction study meticulously analyzed the molecular interactions between the chosen compound and a target protein. CMNPD25830 demonstrated three hydrogen bonds with Arg322, Ser337, and Lys339. It also engaged in nine hydrophobic interactions with Phe363, Glu361, Arg355, Asn429, Ser324, His432, Ser337, Phe363, and Phe404. Furthermore, two π-π stacking interactions were identified with Phe323, Phe325, and His357. The compound CMNPD18675 formed hydrogen bonds with Ser337 and engaged in hydrophobic interactions with Ser324, Ser337, Glu361, Phe363, Phe404, Asn429, Arg332, Arg355, and Val338. CMNPD18676 demonstrated hydrophobic interactions with Asn429, His432, Phe325, Ser324, Phe40, Val338, Ser337, Phe363, Lys331, Ser322, and Met431. Additionally, two π-π stacking interactions were observed with Arg355 and Arg332. CMNPD27216 exhibited two hydrogen bonds with Glu361 and engaged in hydrophobic interactions with Arg332, Ser337, Phe363, Lys376, Phe404, Asn429, Arg355, and Val338. A single π-π stacking interaction was also noted with Phe323, His357 with two bonds, and Phe325. The control 93G showed two hydrogen bonds with Ser324 and Glu361, along with seven hydrophobic interactions with His432, Phe325, Arg33, Ser337, Phe363, Lys376, and Phe404. It also exhibited a π-π stacking interaction (Table 1, Figs 1 and 2). This comprehensive analysis provided valuable insights into the molecular interactions of these compounds.

**Table 1:** Intermolecular interaction of four complexes in complex with the protein CMNPD25830 (b) CMNPD18675 (c) CMNPD18676 (d) CMNPD27216 and (e) Control (93G).

S. no.	Compounds	H-Bond	Compound residue name	Bond length (Å)	π-π interactions	Compound residue name	Bond length (Å)
1	CMNPD25830	Arg^332^ Ser^337^ Lys^339^	C-O, N-H, C-O	1.23266 1.00978 1.22589	Phe^323^ Phe^325^ His^357^	N-H, HA-CA, CA-C, CA-CB	1.01184, 1.09192, 1.5418, 1.52325
2	CMNPD18675	Ser^337^	N-H	1.0096	--	--	--
3	CMNPD18676	--	--	--	Phe^323^ His^357^	CA-C, CA-CB	1.54177, 1.52325
4	CMNPD27216	Glu^361^	N-H	1.01337	Phe^323^ Phe^325^ His^357^	N-H, CA-C, HA-CA	1.01157, 1.5418, 1.08976
5	Control (93G)	Glu^361^ Ser^324^	N-H C-O	1.0099 1.23589	Phe^323^	CA-C	1.54177

### 3.3 MD simulation

Molecular dynamics (MD) simulations are instrumental in shedding light on the dynamic stability of protein-ligand complexes, providing an in-depth understanding of time-dependent molecular interactions. To further our knowledge of the binding interactions and mechanistic actions of four chosen compounds with a target protein, we carried out a comprehensive MD simulation spanning 500 nanoseconds.

#### 3.3.1 Root mean square deviation analysis

**Fig 3:**
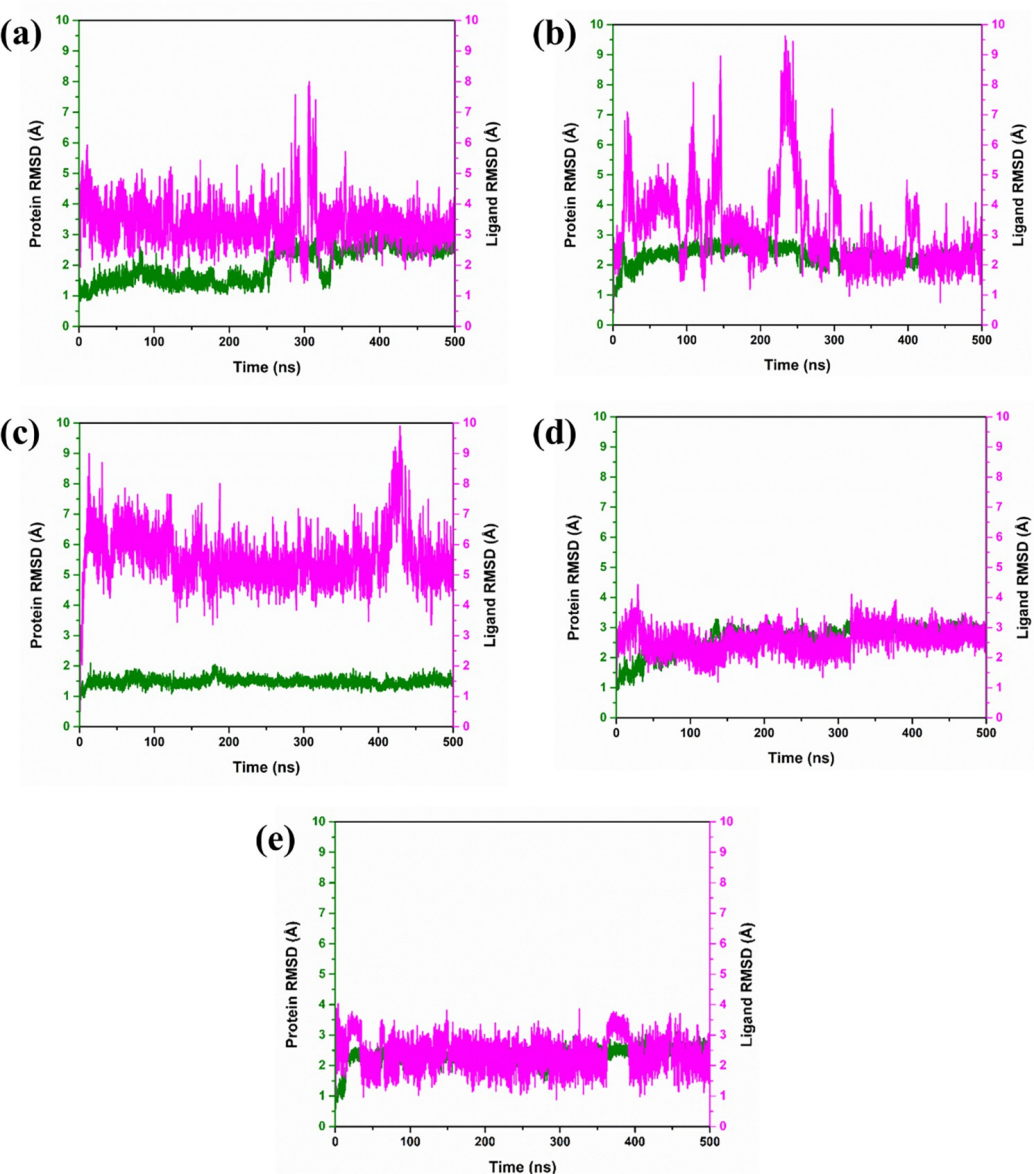
RMSD graph of the selected complexes with the target influenza A H5N1 and compounds i.e., (a) CMNPD25830 (b) CMNPD18675 (c) CMNPD18676 (d) CMNPD27216 and (e) Control during the 500ns simulation.

The root-mean-square deviation (RMSD) analyses for the complexes were conducted based on the results derived from the molecular dynamics simulations. The complex PB2_CMNPD25830 exhibited protein RMSD of 3Å with significant fluctuations during the 200-350ns period and the ligand showed the highest fluctuations of 8 Å around 300ns, after which it stabilized to an RMSD of 4 Å until the end of the simulation. The complex PB2_CMNPD18675 showed high fluctuations in the initial 300ns with an RMSD value of 9 Å, which later stabilized to an RMSD of 4 Å, while the protein remained constant with an RMSD value of 3 Å. The protein RMSD of complex PB2_CMNPD18676 was observed to have a ligand RMSD value of 10 Å with significant fluctuations, implying a potential displacement from the binding pocket, while the protein maintained a constant RMSD of 2 Å. The complex PB2_CMNPD27216 showed significant binding throughout the simulation with a protein RMSD of around 3 Å and ligand around 4 Å, with considerable fluctuations showing the stable binding in the pocket. Lastly, PB2_93G with significant fluctuations of 3–4 Å and protein RMSD of 3 Å suggesting a stable behavior (Fig 3).

#### 3.3.2 Root mean square fluctuation analysis

**Fig 4:**
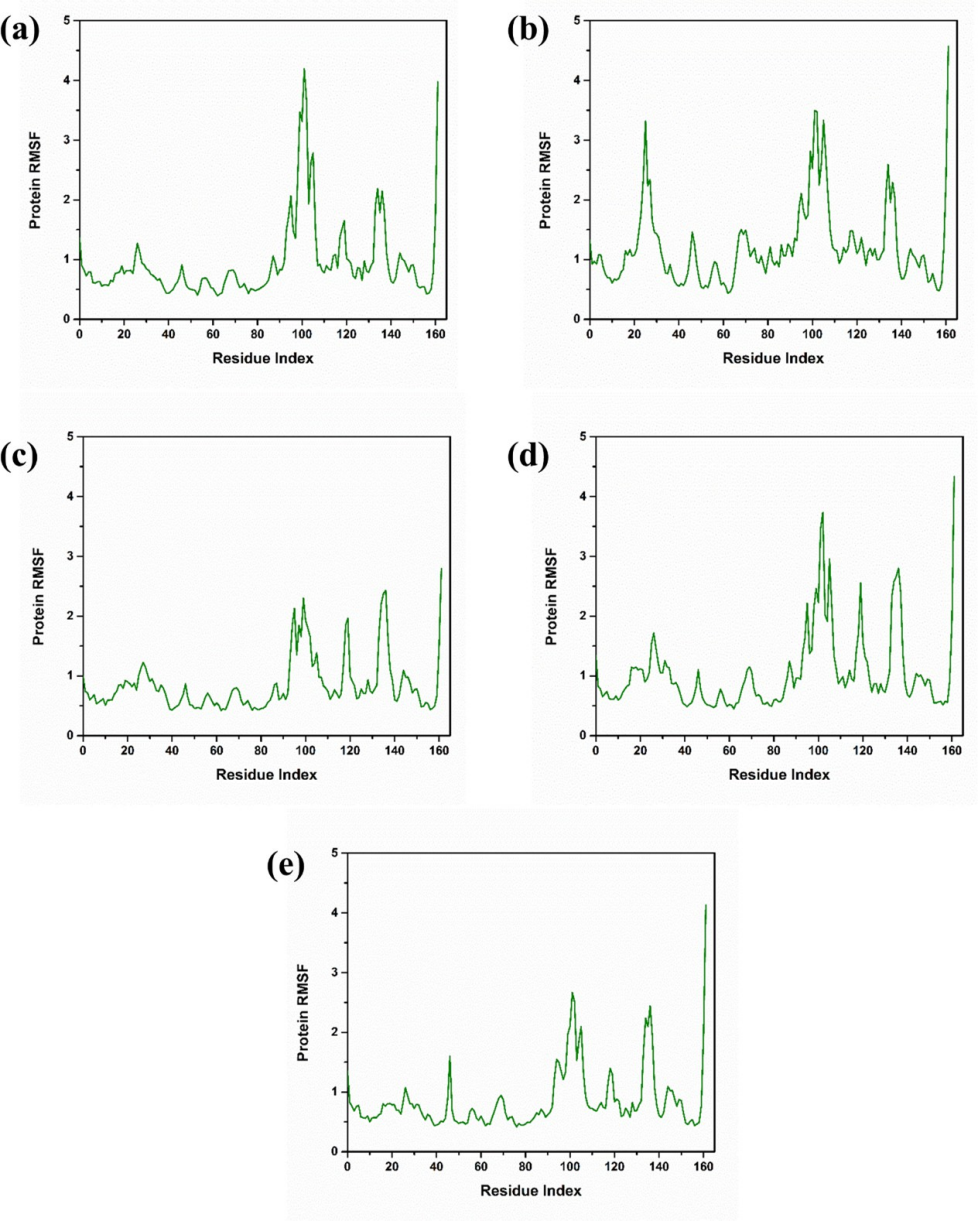
Protein RMSF graph of the selected complexes with the target influenza A H5N1 and compounds i.e., (a) CMNPD25830 (b) CMNPD18675 (c) CMNPD18676 (d) CMNPD27216 and (e) Control during the 500ns simulation.

The Root-Mean-Square Fluctuation (RMSF) analysis conducted on four compounds and a control molecule uncovers a variety of flexibility patterns across distinct segments of the protein residues. complex PB2_CMNPD25830 exhibited significant flexibility, especially at the residue index 100, where the RMSF value surpassed 4Å. In other segments, specifically between indices 20–30 and 100–110, the fluctuations were considerable, recording above 3Å and 3.5Å respectively. The flexibility slightly reduced between indices 130–140, around 2.5Å, but it increased again to more than 4Å at index 160. The complex PB2_CMNPD18675 displayed fluctuation levels of approximately 2.5Å in the regions between indices 90–100 and 130–140, with marginally lower values, just over 2Å, observed between indices 110–120. The complex PB2_CMNPD18676 showed a lesser degree of flexibility in comparison to PB2_CMNPD18675, with the highest fluctuations noted below 4Å between indices 90–100, and staying below 3Å across indices 110–120 and 130–140. Among the compounds, PB2_CMNPD27216 exhibited the smallest fluctuation, with RMSF values falling below 3Å between indices 90–100, decreasing to less than 1.5Å between 110–120, and approximating 2.5Å at indices 130–140. Complex with control PB2_93G demonstrated significant flexibility at a specific site, exceeding 4Å at residual index 100. In other critical regions, the fluctuations resembled those observed in PB2_CMNPD25830, with values above 3Å and 3.5Å between residual indices 20–30 and 100–110 respectively, and peaking at indices 130–140 and 160 residues (Fig 4).

#### 3.3.3 Radius of Gyration (RG)

**Fig 5:**
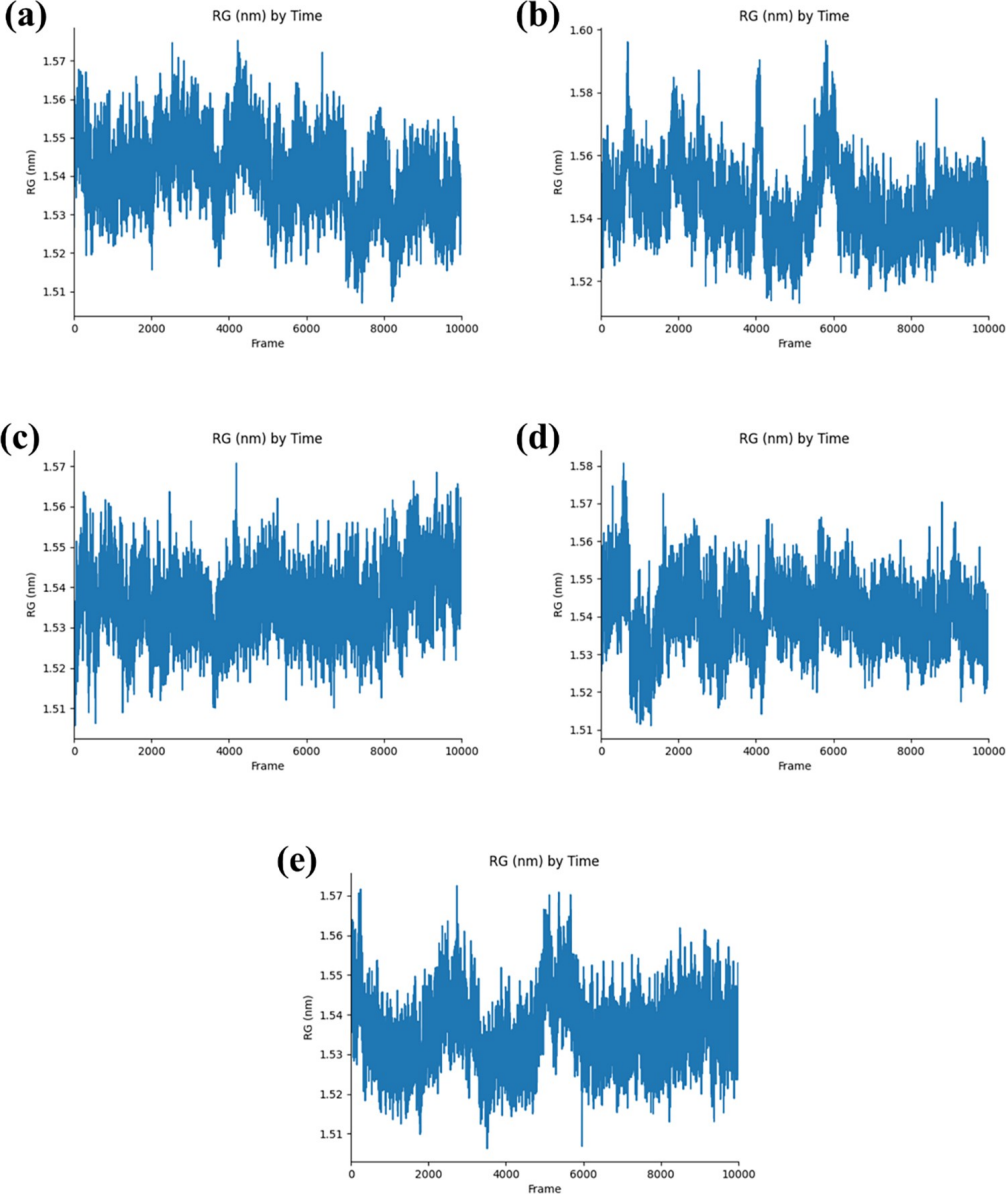
Radius of gyration plots generated for the selected complexes with the target influenza A H5N1 and compounds i.e., (a) CMNPD25830 (b) CMNPD18675 (c) CMNPD18676 (d) CMNPD27216 and (e) Control during the 500ns simulation.

The RG of PB2_CMNPD25830 complex initially showed fluctuations within a confined range of 1.54 nm to 1.55 nm for about 7000 frames. Following this, the RG value settled to a slightly lower constant value around 1.53 nm. The PB2_CMNPD18675 displayed considerable fluctuations in the radius of gyration up to the 6000th frame. After this period of instability, the RG value reached a steady state at approximately 1.54 nm, indicating the molecular structure’s stabilization as the simulation advanced. Complex PB2_CMNPD18676 consistently maintained an RG with significant fluctuations fluctuation between 1.53 nm and 1.54 nm throughout the simulation. This behavior suggests a dynamic balance within the molecular structure, with no noticeable settling. The PB2_CMNPD27216 exhibited substantial fluctuations at the beginning of the simulation, reaching up to 1.58 nm. After this initial period of instability, the radius of gyration decreased and then sustained stable peaks around 1.56 nm for the rest of the simulation, indicating a shift back to a more stable molecular conformation post the initial disruptions. Control with protein complexed, PB2_93G began with an RG of 1.57 nm and experienced significant deviations during the simulation’s first half. These deviations gradually reduced, and the radius of gyration stabilized at 1.56 nm, suggesting that the system achieved a steady state later in the simulation. This comparative analysis indicates that although all compounds undergo fluctuations, the magnitude and timing of these fluctuations differ. PB2_CMNPD25830 and PB2_CMNPD18675 attain a stable and denser state more rapidly than PB2_93G, which, together with PB2_CMNPD27216, shows more noticeable initial deviations but ultimately reaches stability (Fig 5).

#### 3.3.4 Hydrogen bond

**Fig 6:**
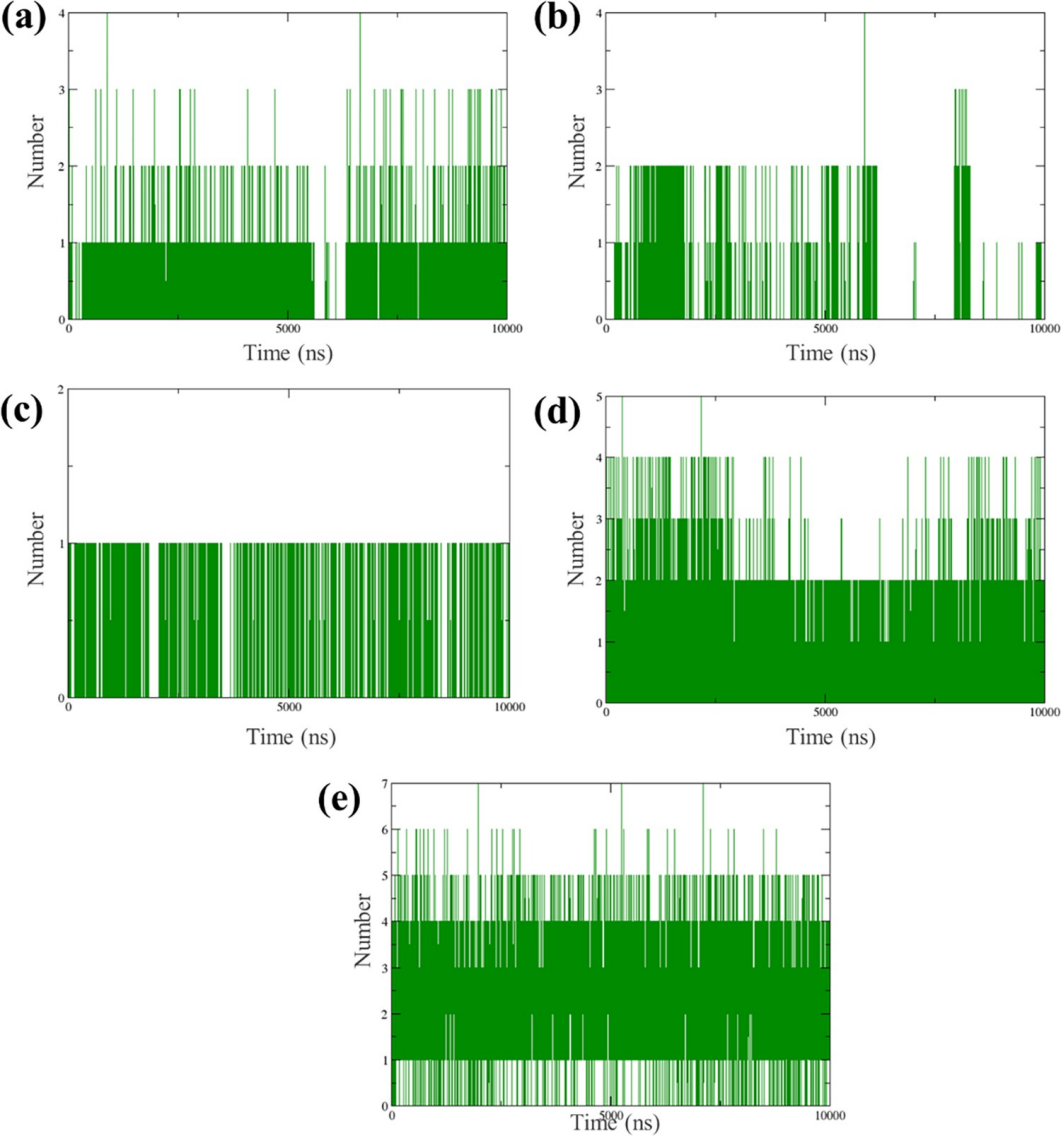
Hydrogen bond generated for the selected complexes with the target influenza A H5N1 and compounds i.e., (a) CMNPD25830 (b) CMNPD18675 (c) CMNPD18676 (d) CMNPD27216 and (e) Control during the 500ns simulation.

In the simulation, PB2_CMNPD25830 initially maintained 2–3 hydrogen bonds up to 5000 frames, after which bond dissociation occurred. A more stable configuration of 2 hydrogen bonds was established around the 6000th frame and maintained until the simulation, suggesting an initial dynamic phase followed by stabilization in the hydrogen bonding pattern. The complex PB2_CMNPD18675 started with 2 hydrogen bonds, consistent up to the first 5000 frames. However, some of these bonds broke afterwards, leading to a reduced bonding configuration of only one hydrogen bond towards the end of the simulation, indicating a decrease in molecular interaction stability over time. The complex PB2_CMNPD18676 exhibited a single hydrogen bond throughout the simulation, indicating a consistent but potentially less robust interaction, as multiple bonds typically suggest stronger or more stable interactions. The PB2_CMNPD27216 initially had more than 4 hydrogen bonds, indicating a high level of interaction. By the end of the simulation, it maintained 2 stable hydrogen bonds, suggesting a reduction but eventual stabilization in the number of significant interactions. Lastly, PB2_93G showed a high number of hydrogen bonds, starting with more than 6, and maintaining 3–4 stable bonds throughout the simulation. The complexes PB2_CMNPD25830 and PB2_CMNPD27216 eventually match the stability of the control, demonstrating their good adaptability. On the other hand, PB2_CMNPD18675 and PB2_CMNPD18676 exhibit less interaction strength and stability, which could potentially impact their functionality, depending on the molecular interaction needs of their intended uses (Fig 6).

### 3.4 Binding free energy analysis

The total free binding energy (ΔG_bind) from molecular dynamics simulations observed a range of binding affinities across different complexes and the control. The complex PB2_CMNPD25830, and PB2_CMNPD18675 displayed slightly less favorable binding energies of -45.5±14.71 kcal/mol -44.28±13.94 kcal/mol, respectively. The least favorable binding was seen in complex PB2_CMNPD18676, with a ΔG_bind of -38.87±9.41 kcal/mol. The complex PB2_CMNPD27216 demonstrated the most favorable binding with the lowest ΔG_bind of -57.14±13.57 kcal/mol, indicating strong binding interactions. This was closely followed by the control compound PB2_Control (93G), which exhibited a ΔG_bind of -59.71±14.34 kcal/mol, suggesting similarly strong binding interactions. These findings indicate a variety of binding efficiencies among the compounds, with PB2_CMNPD27216 and the control showing the greatest potential for stable interactions based on their lower ΔG_bind values (Table 2).

**Table 2:** MM/GBSA table for the selected complexes i.e., (a) CMNPD25830 (b) CMNPD18675 (c) CMNPD18676 (d) CMNPD27216 and (e) Control.

Energy components/ Complexes	PB2_CMNPD25830	PB2_CMNPD18675	PB2_ CMNPD18676	PB2_CMNPD27216	PB2_Control (93G)
Van der Waal energy (ΔVDWAALS)	-34.79±3.15	-49.57±4.43	-41.20±3.09	-49.85±3.22	-43.66±3.04
Electrostatic energy(ΔEEL)	-21.15±4.18	-7.18±5.78	-3.17±4.03	-17.71±5.50	-27.94±6.57
Polar solvation energy (ΔEGB)	-25.45±4.22	31.74±3.0	20.5±4.8	30.16±3.10	25.15±3.89
Non-polar solvation energy (ΔESURF)	-13.78±1.73	-20.48±1.47	-15.0±1.49	-19.74±1.73	-13.25±0.83
Net gas phase energy (ΔGGAS)	-55.95±7.97	-56.76±10.22	-44.38±3.09	-67.57±8.73	-71.61±9.61
Net solvation energy (ΔGSOLV)	11.66±5.96	11.26±4.48	5.50±6.31	10.42±4.83	11.89±4.72
ΔG_total_	-44.28±13.94	-45.5±14.71	-38.87±9.41	-57.14±13.57	-59.71±14.34

### 3.5 RG-RMSD-based free energy landscape

**Fig 7:**
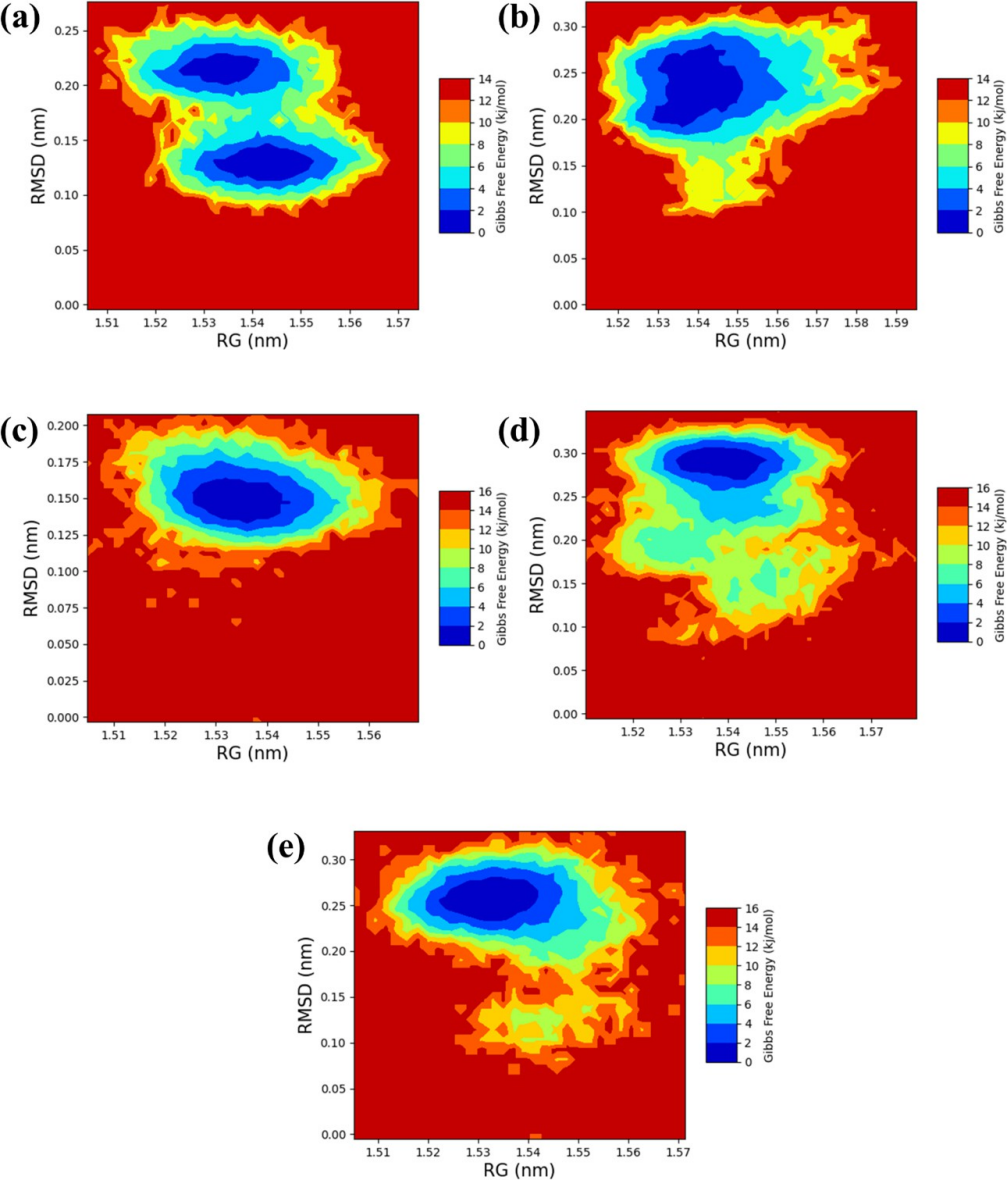
2D plots of the free energy landscape of the selected complexes with the target influenza A H5N1 and compounds i.e., (a) CMNPD25830 (b) CMNPD18675 (c) CMNPD18676 (d) CMNPD27216 and (e) Control.

The free energy landscapes, based on the radius of gyration (Rg), offer a comprehensive assessment of the structural compactness and stability of complexes interacting with the influenza A H5N1 PB2 binding protein. The Complex PB2_CMNPD25830 and PB2_CMNPD18675 might suggest less stable binding configurations due to fewer or shallower energy wells. This is inferred from broader areas with higher energy levels, indicating a tendency for these complexes to explore a wider range of conformations without a strong preference for a single, highly stable state. On the other hand, Complex PB2_CMNPD18676 could have a stable yet dynamically accessible binding mode if its landscape shows a few deep wells and lower barriers. This characteristic is beneficial for interactions where the protein needs to undergo conformational changes as part of its function. Complex PB2_CMNPD27216, if it demonstrates the deepest and most numerous energy wells, would suggest a very stable interaction with the protein, offering multiple favourable binding modes. This could make it a strong candidate for therapeutic development if these stable states are associated with effective inhibition of the protein’s function. Lastly, the landscape of the control, PB2_Control 93G, serves as a standard for comparison. If it exhibits moderate well depth and barriers, any compound with deeper and more numerous wells would be considered to have enhanced interaction characteristics (Fig 7 and S1 Fig in S1 File).

Overall analysis suggests that CMNPD25830, CMNPD18676, and CMNPD27216 demonstrate more stable binding to the influenza A H5N1 target, characterized by distinct low-energy basins that suggest a potential for high efficacy. In contrast, the landscape for the control is notably less favourable, suggesting that these studied compounds are superior in their interaction with the H5N1 target, possibly offering enhanced therapeutic benefits.

**Fig 8:**
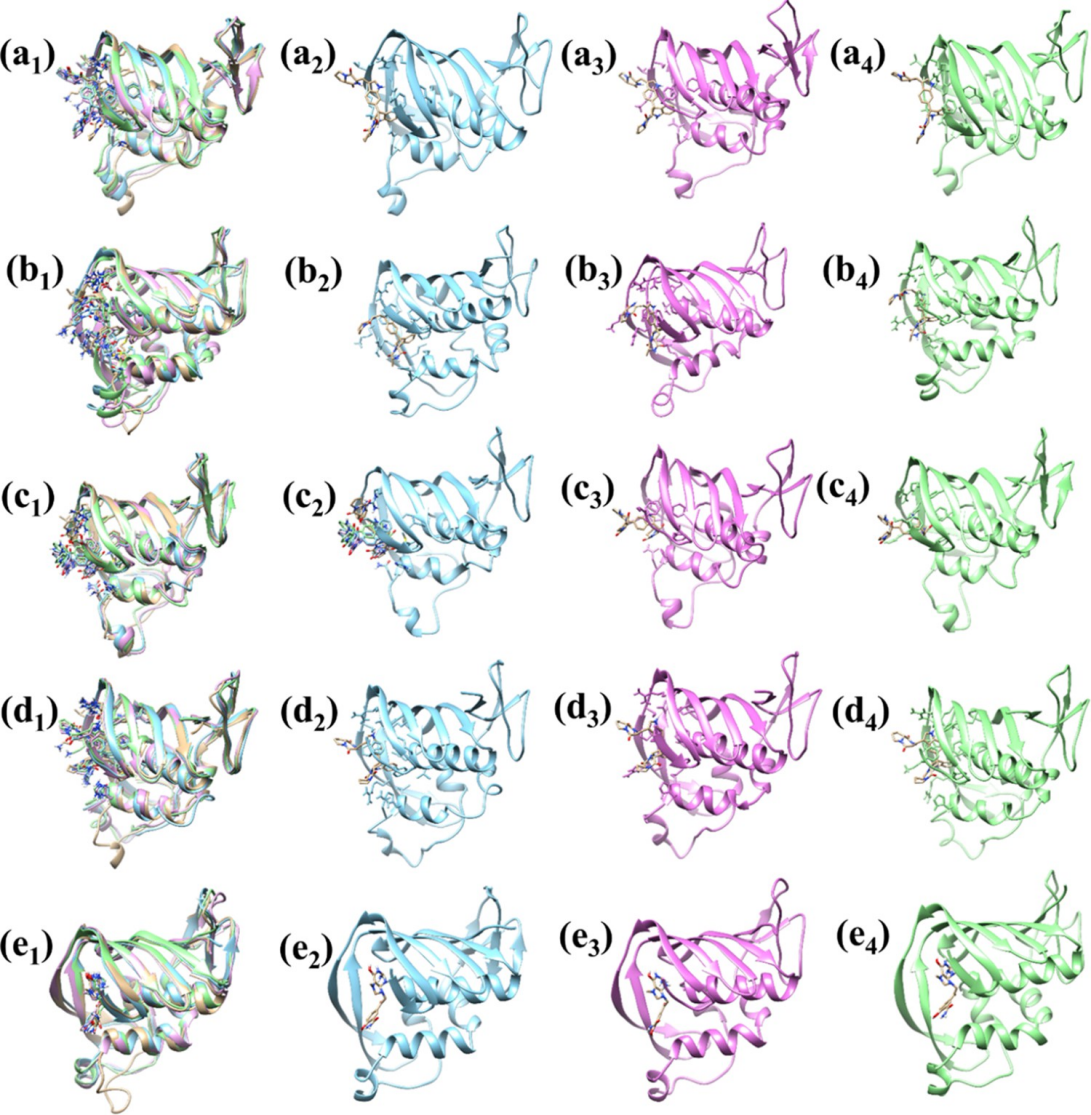
Superimposed representation of the selected complexes with the target influenza A H5N1 and compounds i.e., (a_1_-a_2_) CMNPD25830 (b_1_-b_2_) CMNPD18675 (c_1_-c_2_) CMNPD18676 (d_1_-d_2_) CMNPD27216 and (e_1_-e_2_) Control.

The superimposition of the complexes with the control provides a comparative analysis of the structural behaviour among the compounds. The superimposition is quantified by the RMSD values post-superimposition, which measure the average distance between the atoms of the superimposed molecules. The complex PB2_CMNPD25830 shows an RMSD of 1.22 Å, indicating a slight deviation from the control’s structure which exhibits an RMSD of 1.16 Å. The complex PB2_CMNPD18675 and PB2_CMNPD18676 observed 1.45 and 1.07 Å, respectively, indicating more significant deviation from the control compound while the PB2_CMNPD18676 suggests minimal structural displacement. The PB2_CMNPD27216 complex showed an RMSD of 1.20 Å suggesting a slightly altered conformation closely resembling the control. The analysis is crucial for comprehending the structural basis of compound efficacy and directing subsequent alternations to enhance therapeutic efficiency (Fig 8).

## 4. Discussion

The research has shed light on the potential of molecules derived from marine bacteria to inhibit the influenza polymerase PB2 cap-binding domain. The study emphasized a comparative analysis with the control molecule, (93G). Four compounds (CMNPD25830, CMNPD18675, CMNPD18676, and CMNPD27216) emerged as promising candidates during virtual screening, owing to their high binding energies that were either superior or comparable to the control molecule. Further analysis through re-docking and molecular dynamics (MD) simulations unveiled subtle differences in the stability and interaction dynamics of these compounds within the PB2 binding site. Notably, CMNPD27216 and CMNPD25830 exhibited binding energies and interaction profiles that were, in some respects, superior to the control molecule. For instance, CMNPD27216 demonstrated a strong binding affinity, as indicated by a favorable ΔG_bind of -57.14 kcal/mol, which is slightly less than the control’s -59.71 kcal/mol, suggesting potent inhibitory capabilities. MD simulations further revealed that CMNPD27216 and CMNPD25830 maintained more stable hydrogen bonding configurations compared to the control, a critical factor for effective inhibition. These compounds also showed lower root-mean-square deviation (RMSD) values during the simulations, indicating a more stable interaction with the PB2 binding site over time. The robustness of these interactions was further substantiated by free energy landscape analysis. CMNPD27216, in particular, exhibited numerous and deep energy wells, indicative of a variety of stable binding conformations, which are beneficial for effective inhibition. This suggests that CMNPD27216 not only mimics the control in terms of binding affinity but may also provide alternative binding modes that could be exploited to circumvent resistance mechanisms.

In 2013, Pautus and his team conducted a study using docking studies to discover potential inhibitors. They identified 7-alkylguanine derivatives, specifically substituted at N-9 and N-2 positions, as promising candidates [18]. Zhao et al.’s 2020 study revealed the identification of novel inhibitors that target the PB2 cap-binding domain, a significant stride in the advancement of antiviral therapies against influenza. Four compounds 11D4, 12C5, 21A5, and 21B1 were identified from the ChemBridge library [12]. Another study by Zong et al., (2021), demonstrated the effectiveness of certain inhibitors. The study employed docking studies and proposed that 7-alkylguanine derivatives, specifically substituted at the N-9 and N-2 positions, could serve as powerful inhibitors [48]. In comparison to a recently published study, which identified four lead compounds (ZINC000096095464, ZINC000044404209, ZINC000001562130, ZINC000059779788) with binding energies of -9.6, -9.4, -9.3, and -9.2 Kcal/mol, our findings demonstrate superior binding affinities [49]. This indicates that our identified compounds not only possess stronger molecular interactions with the target protein but also hold greater potential for further development. The improved binding energies observed in our study highlight the enhanced efficacy of our lead compounds, suggesting they could be more promising candidates for future drug design and optimization.

## 5. Conclusion

This study employed an extensive computational drug discovery approach to identify the potential inhibitors of the Influenza A H5N1 PB2 cap-binding domain in marine bacteria. The comparative analysis identifies CMNPD27216 and CMNPD25830 as potential anti-influenza drugs worthy of additional investigation and development. The findings underscore the importance of marine bacteria as a novel source of antiviral drugs and highlight the necessity of detailed molecular dynamics studies for validating and understanding the behaviour of potential therapeutic candidates in a dynamic biological context. The study’s outcomes could potentially pave the way for the creation of novel and effective antiviral therapies against the influenza virus. The next crucial phase in this journey would be further experimental validation of the potential inhibitors identified, a critical step in their advancement through the drug development pipeline.

## Supporting information

S1 File (DOCX)
