# Supplementary material for: Investigating Pb2 CAP-binding domain inhibitors from marine bacteria for targeting the influenza A H5N1
Source: PLoS One. 2025 Jan 28;20(1):e0310836. doi: 10.1371/journal.pone.0310836 (PMC11774345; doi:10.1371/journal.pone.0310836)
Supplement: S1 File — (DOCX) [file pone.0310836.s001.docx]

**S1 Table -** List of Marine Bacteria compounds library screened on MTi-Autodock.

| **Compounds** | **Docking Energy (kcal/mol)** |
| --- | --- |
| CMNPD25830 | -11.3 |
| CMNPD18675 | -11.1 |
| CMNPD18676 | -11 |
| CMNPD27216 | -10.9 |
| CMNPD27283 | -10.8 |
| CMNPD30132 | -10.3 |
| CMNPD21065 | -10.3 |
| CMNPD21066 | -10.2 |
| CMNPD25859 | -10.1 |
| CMNPD30010 | -10.1 |
| CMNPD15686 | -10 |
| CMNPD21969 | -10 |
| CMNPD30135 | -9.9 |
| CMNPD25857 | -9.9 |
| CMNPD30138 | -9.9 |
| CMNPD25780 | -9.9 |
| CMNPD24329 | -9.8 |
| CMNPD30144 | -9.8 |
| CMNPD21933 | -9.8 |
| CMNPD30151 | -9.7 |
| CMNPD19652 | -9.7 |
| CMNPD17558 | -9.7 |
| CMNPD25797 | -9.7 |
| CMNPD30143 | -9.7 |
| CMNPD14698 | -9.6 |
| CMNPD18900 | -9.6 |
| CMNPD30133 | -9.6 |
| CMNPD10129 | -9.6 |
| CMNPD24377 | -9.6 |
| CMNPD21931 | -9.6 |
| CMNPD30137 | -9.6 |
| CMNPD14710 | -9.6 |
| CMNPD30136 | -9.6 |
| CMNPD30141 | -9.5 |
| CMNPD20787 | -9.5 |
| CMNPD24524 | -9.5 |
| CMNPD9393 | -9.5 |
| CMNPD25863 | -9.4 |
| CMNPD10130 | -9.4 |
| CMNPD28576 | -9.4 |
| CMNPD19681 | -9.4 |
| CMNPD23223 | -9.4 |
| CMNPD16562 | -9.4 |
| CMNPD25806 | -9.4 |
| CMNPD16561 | -9.3 |
| CMNPD28575 | -9.3 |
| CMNPD24480 | -9.3 |
| CMNPD30139 | -9.3 |
| CMNPD15701 | -9.3 |
| CMNPD24402 | -9.3 |
| CMNPD30140 | -9.3 |
| CMNPD25858 | -9.3 |
| CMNPD27163 | -9.3 |
| CMNPD19675 | -9.3 |
| CMNPD27164 | -9.2 |
| CMNPD19659 | -9.2 |
| CMNPD15702 | -9.2 |
| CMNPD25807 | -9.2 |
| CMNPD15704 | -9.2 |
| CMNPD25939 | -9.2 |
| CMNPD16733 | -9.2 |
| CMNPD25741 | -9.2 |
| CMNPD30145 | -9.2 |
| CMNPD30072 | -9.1 |
| CMNPD30129 | -9.1 |
| CMNPD5875 | -9.1 |
| CMNPD28470 | -9.1 |
| CMNPD21897 | -9.1 |
| CMNPD27138 | -9.1 |
| CMNPD30149 | -9.1 |
| CMNPD28578 | -9.1 |
| CMNPD21971 | -9.1 |
| CMNPD13253 | -9.1 |
| CMNPD19739 | -9.1 |
| CMNPD10907 | -9.1 |
| CMNPD30142 | -9.1 |
| CMNPD30134 | -9.1 |
| CMNPD19682 | -9 |
| CMNPD28444 | -9 |
| CMNPD10900 | -9 |
| CMNPD21932 | -9 |
| CMNPD29944 | -9 |
| CMNPD7236 | -9 |
| CMNPD30019 | -9 |
| CMNPD28509 | -9 |
| CMNPD6531 | -9 |
| CMNPD28406 | -9 |
| CMNPD19740 | -9 |
| CMNPD30130 | -9 |
| CMNPD30073 | -9 |
| CMNPD15703 | -9 |
| CMNPD30067 | -9 |
| CMNPD28445 | -8.9 |
| CMNPD10908 | -8.9 |
| CMNPD19678 | -8.9 |
| CMNPD28413 | -8.9 |
| CMNPD14684 | -8.9 |
| CMNPD27247 | -8.9 |
| CMNPD23213 | -8.9 |
| CMNPD30148 | -8.9 |
| CMNPD27166 | -8.9 |
| CMNPD28577 | -8.9 |
| CMNPD30030 | -8.9 |
| CMNPD30032 | -8.9 |
| CMNPD15676 | -8.9 |
| CMNPD10901 | -8.8 |
| CMNPD13187 | -8.8 |
| CMNPD23163 | -8.8 |
| CMNPD19650 | -8.8 |
| CMNPD8741 | -8.8 |
| CMNPD24356 | -8.8 |
| CMNPD30058 | -8.8 |
| CMNPD30031 | -8.8 |
| CMNPD27165 | -8.8 |
| CMNPD25788 | -8.8 |
| CMNPD23161 | -8.8 |
| CMNPD18611 | -8.8 |
| CMNPD28572 | -8.8 |
| CMNPD30005 | -8.8 |
| CMNPD241 | -8.8 |
| CMNPD240 | -8.8 |
| CMNPD20715 | -8.8 |
| CMNPD20738 | -8.7 |
| CMNPD20698 | -8.7 |
| CMNPD19725 | -8.7 |
| CMNPD19696 | -8.7 |
| CMNPD30131 | -8.7 |
| CMNPD17537 | -8.7 |
| CMNPD25749 | -8.7 |
| CMNPD15675 | -8.7 |
| CMNPD25802 | -8.7 |
| CMNPD21900 | -8.7 |
| CMNPD21899 | -8.7 |
| CMNPD25781 | -8.7 |
| CMNPD30150 | -8.7 |
| CMNPD27169 | -8.7 |
| CMNPD28559 | -8.6 |
| CMNPD20785 | -8.6 |
| CMNPD23263 | -8.6 |
| CMNPD19702 | -8.6 |
| CMNPD30123 | -8.6 |
| CMNPD27134 | -8.6 |
| CMNPD25880 | -8.6 |
| CMNPD11654 | -8.6 |
| CMNPD23212 | -8.6 |
| CMNPD7992 | -8.6 |
| CMNPD30054 | -8.6 |
| CMNPD28408 | -8.6 |
| CMNPD19657 | -8.6 |
| CMNPD1749 | -8.6 |
| CMNPD27170 | -8.6 |
| CMNPD25779 | -8.6 |
| CMNPD20768 | -8.6 |
| CMNPD20714 | -8.6 |
| CMNPD19724 | -8.6 |
| CMNPD30035 | -8.5 |
| CMNPD25866 | -8.5 |
| CMNPD25803 | -8.5 |
| CMNPD21923 | -8.5 |
| CMNPD2188 | -8.5 |
| CMNPD27135 | -8.5 |
| CMNPD13185 | -8.5 |
| CMNPD28598 | -8.5 |
| CMNPD6529 | -8.5 |
| CMNPD16556 | -8.5 |
| CMNPD30018 | -8.5 |
| CMNPD19703 | -8.5 |
| CMNPD18664 | -8.5 |
| CMNPD30068 | -8.5 |
| CMNPD30036 | -8.5 |
| CMNPD30216 | -8.5 |
| CMNPD30053 | -8.5 |
| CMNPD20753 | -8.5 |
| CMNPD10898 | -8.5 |
| CMNPD27783 | -8.5 |
| CMNPD28550 | -8.5 |
| CMNPD25799 | -8.5 |
| CMNPD28570 | -8.4 |
| CMNPD30008 | -8.4 |
| CMNPD14707 | -8.4 |
| CMNPD7280 | -8.4 |
| CMNPD19658 | -8.4 |
| CMNPD21924 | -8.4 |
| CMNPD23169 | -8.4 |
| CMNPD15801 | -8.4 |
| CMNPD9394 | -8.4 |
| CMNPD28638 | -8.4 |
| CMNPD29994 | -8.4 |
| CMNPD6530 | -8.4 |
| CMNPD19680 | -8.4 |
| CMNPD23216 | -8.4 |
| CMNPD25800 | -8.4 |
| CMNPD19727 | -8.4 |
| CMNPD28513 | -8.4 |
| CMNPD19661 | -8.4 |
| CMNPD28510 | -8.4 |
| CMNPD28558 | -8.4 |
| CMNPD17563 | -8.4 |
| CMNPD27136 | -8.4 |
| CMNPD20729 | -8.4 |
| CMNPD21903 | -8.4 |
| CMNPD24527 | -8.4 |
| CMNPD19896 | -8.4 |
| CMNPD28487 | -8.4 |
| CMNPD24376 | -8.4 |
| CMNPD21898 | -8.3 |
| CMNPD18865 | -8.3 |
| CMNPD28517 | -8.3 |
| CMNPD24515 | -8.3 |
| CMNPD30037 | -8.3 |
| CMNPD28411 | -8.3 |
| CMNPD13262 | -8.3 |
| CMNPD24398 | -8.3 |
| CMNPD28467 | -8.3 |
| CMNPD19677 | -8.3 |
| CMNPD21970 | -8.3 |
| CMNPD30049 | -8.3 |
| CMNPD23194 | -8.3 |
| CMNPD24471 | -8.3 |
| CMNPD19729 | -8.3 |
| CMNPD21960 | -8.3 |
| CMNPD25798 | -8.3 |
| CMNPD19722 | -8.3 |
| CMNPD25729 | -8.3 |
| CMNPD30217 | -8.3 |
| CMNPD24347 | -8.3 |
| CMNPD21926 | -8.3 |
| CMNPD19660 | -8.3 |
| CMNPD27769 | -8.3 |
| CMNPD1761 | -8.3 |
| CMNPD19662 | -8.3 |
| CMNPD28405 | -8.3 |
| CMNPD23298 | -8.3 |
| CMNPD23250 | -8.3 |
| CMNPD28453 | -8.3 |
| CMNPD19728 | -8.2 |
| CMNPD20731 | -8.2 |
| CMNPD16564 | -8.2 |
| CMNPD25801 | -8.2 |
| CMNPD23211 | -8.2 |
| CMNPD3607 | -8.2 |
| CMNPD20718 | -8.2 |
| CMNPD24500 | -8.2 |
| CMNPD28511 | -8.2 |
| CMNPD28414 | -8.2 |
| CMNPD28407 | -8.2 |
| CMNPD13996 | -8.2 |
| CMNPD23249 | -8.2 |
| CMNPD5307 | -8.2 |
| CMNPD30048 | -8.2 |
| CMNPD11649 | -8.2 |
| CMNPD23311 | -8.2 |
| CMNPD23265 | -8.2 |
| CMNPD24384 | -8.2 |
| CMNPD24369 | -8.2 |
| CMNPD28488 | -8.2 |
| CMNPD27110 | -8.2 |
| CMNPD27214 | -8.2 |
| CMNPD5309 | -8.2 |
| CMNPD24408 | -8.2 |
| CMNPD13191 | -8.2 |
| CMNPD18628 | -8.2 |
| CMNPD6532 | -8.2 |
| CMNPD28571 | -8.2 |
| CMNPD10899 | -8.2 |
| CMNPD20786 | -8.2 |
| CMNPD30017 | -8.2 |
| CMNPD21942 | -8.2 |
| CMNPD19699 | -8.2 |
| CMNPD28512 | -8.2 |
| CMNPD12464 | -8.2 |
| CMNPD21965 | -8.2 |
| CMNPD18877 | -8.2 |
| CMNPD21025 | -8.2 |
| CMNPD17544 | -8.2 |
| CMNPD18881 | -8.2 |
| CMNPD30070 | -8.2 |
| CMNPD20783 | -8.1 |
| CMNPD19897 | -8.1 |
| CMNPD25752 | -8.1 |
| CMNPD24366 | -8.1 |
| CMNPD21966 | -8.1 |
| CMNPD21948 | -8.1 |
| CMNPD27137 | -8.1 |
| CMNPD21925 | -8.1 |
| CMNPD24401 | -8.1 |
| CMNPD3637 | -8.1 |
| CMNPD19701 | -8.1 |
| CMNPD24350 | -8.1 |
| CMNPD28671 | -8.1 |
| CMNPD30153 | -8.1 |
| CMNPD27120 | -8.1 |
| CMNPD16583 | -8.1 |
| CMNPD25787 | -8.1 |
| CMNPD19895 | -8.1 |
| CMNPD24969 | -8.1 |
| CMNPD30125 | -8.1 |
| CMNPD20866 | -8.1 |
| CMNPD24426 | -8.1 |
| CMNPD28646 | -8.1 |
| CMNPD28613 | -8.1 |
| CMNPD13192 | -8.1 |
| CMNPD30001 | -8.1 |
| CMNPD15777 | -8.1 |
| CMNPD27121 | -8.1 |
| CMNPD23168 | -8.1 |
| CMNPD27168 | -8.1 |
| CMNPD23222 | -8.1 |
| CMNPD23217 | -8.1 |
| CMNPD5858 | -8.1 |
| CMNPD20695 | -8.1 |
| CMNPD21941 | -8.1 |
| CMNPD28501 | -8.1 |
| CMNPD24395 | -8.1 |
| CMNPD330 | -8.1 |
| CMNPD27167 | -8.1 |
| CMNPD18663 | -8.1 |
| CMNPD24330 | -8.1 |
| CMNPD24962 | -8.1 |
| CMNPD19733 | -8.1 |
| CMNPD20782 | -8 |
| CMNPD21022 | -8 |
| CMNPD18880 | -8 |
| CMNPD20746 | -8 |
| CMNPD16567 | -8 |
| CMNPD19898 | -8 |
| CMNPD24468 | -8 |
| CMNPD18876 | -8 |
| CMNPD18680 | -8 |
| CMNPD1748 | -8 |
| CMNPD7990 | -8 |
| CMNPD24367 | -8 |
| CMNPD25796 | -8 |
| CMNPD30029 | -8 |
| CMNPD27221 | -8 |
| CMNPD17578 | -8 |
| CMNPD7989 | -8 |
| CMNPD19649 | -8 |
| CMNPD28469 | -8 |
| CMNPD20697 | -8 |
| CMNPD23215 | -8 |
| CMNPD21892 | -8 |
| CMNPD23228 | -8 |
| CMNPD27245 | -8 |
| CMNPD15699 | -8 |
| CMNPD13886 | -8 |
| CMNPD30020 | -8 |
| CMNPD24351 | -8 |
| CMNPD28676 | -8 |
| CMNPD29943 | -8 |
| CMNPD28450 | -8 |
| CMNPD30189 | -8 |
| CMNPD27791 | -8 |
| CMNPD27188 | -8 |
| CMNPD24400 | -8 |
| CMNPD15776 | -8 |
| CMNPD341 | -8 |
| CMNPD27275 | -8 |
| CMNPD28642 | -8 |
| CMNPD24526 | -8 |
| CMNPD19695 | -8 |
| CMNPD29954 | -8 |
| CMNPD17561 | -8 |
| CMNPD12472 | -8 |
| CMNPD25817 | -8 |
| CMNPD7991 | -8 |
| CMNPD21901 | -8 |
| CMNPD23226 | -8 |
| CMNPD20696 | -8 |
| CMNPD23191 | -8 |
| CMNPD1759 | -8 |
| CMNPD19679 | -8 |
| CMNPD20719 | -8 |
| CMNPD23203 | -7.9 |
| CMNPD21036 | -7.9 |
| CMNPD335 | -7.9 |
| CMNPD30090 | -7.9 |
| CMNPD8747 | -7.9 |
| CMNPD29998 | -7.9 |
| CMNPD25784 | -7.9 |
| CMNPD6536 | -7.9 |
| CMNPD12467 | -7.9 |
| CMNPD20784 | -7.9 |
| CMNPD27228 | -7.9 |
| CMNPD20720 | -7.9 |
| CMNPD7284 | -7.9 |
| CMNPD25737 | -7.9 |
| CMNPD28614 | -7.9 |
| CMNPD13263 | -7.9 |
| CMNPD30046 | -7.9 |
| CMNPD13254 | -7.9 |
| CMNPD5300 | -7.9 |
| CMNPD23252 | -7.9 |
| CMNPD28553 | -7.9 |
| CMNPD17576 | -7.9 |
| CMNPD24430 | -7.9 |
| CMNPD23190 | -7.9 |
| CMNPD24433 | -7.9 |
| CMNPD23162 | -7.9 |
| CMNPD28536 | -7.9 |
| CMNPD28605 | -7.9 |
| CMNPD28452 | -7.9 |
| CMNPD21895 | -7.9 |
| CMNPD20867 | -7.9 |
| CMNPD30044 | -7.9 |
| CMNPD25745 | -7.9 |
| CMNPD20744 | -7.9 |
| CMNPD28410 | -7.9 |
| CMNPD24348 | -7.9 |
| CMNPD19687 | -7.9 |
| CMNPD21961 | -7.9 |
| CMNPD21962 | -7.9 |
| CMNPD13300 | -7.9 |
| CMNPD30012 | -7.9 |
| CMNPD18668 | -7.9 |
| CMNPD17553 | -7.9 |
| CMNPD27210 | -7.9 |
| CMNPD874 | -7.9 |
| CMNPD20722 | -7.9 |
| CMNPD27246 | -7.9 |
| CMNPD23207 | -7.9 |
| CMNPD25876 | -7.9 |
| CMNPD21902 | -7.9 |
| CMNPD5311 | -7.9 |
| CMNPD334 | -7.9 |
| CMNPD30016 | -7.9 |
| CMNPD30034 | -7.9 |
| CMNPD22327 | -7.9 |
| CMNPD25768 | -7.8 |
| CMNPD24483 | -7.8 |
| CMNPD28557 | -7.8 |
| CMNPD24502 | -7.8 |
| CMNPD17549 | -7.8 |
| CMNPD19734 | -7.8 |
| CMNPD30126 | -7.8 |
| CMNPD24459 | -7.8 |
| CMNPD24484 | -7.8 |
| CMNPD15697 | -7.8 |
| CMNPD16712 | -7.8 |
| CMNPD5306 | -7.8 |
| CMNPD21957 | -7.8 |
| CMNPD23147 | -7.8 |
| CMNPD23239 | -7.8 |
| CMNPD18867 | -7.8 |
| CMNPD18869 | -7.8 |
| CMNPD5308 | -7.8 |
| CMNPD9391 | -7.8 |
| CMNPD16569 | -7.8 |
| CMNPD17532 | -7.8 |
| CMNPD27140 | -7.8 |
| CMNPD14708 | -7.8 |
| CMNPD30057 | -7.8 |
| CMNPD17550 | -7.8 |
| CMNPD3602 | -7.8 |
| CMNPD19664 | -7.8 |
| CMNPD18864 | -7.8 |
| CMNPD30007 | -7.8 |
| CMNPD30047 | -7.8 |
| CMNPD24461 | -7.8 |
| CMNPD30062 | -7.8 |
| CMNPD25794 | -7.8 |
| CMNPD26425 | -7.8 |
| CMNPD23256 | -7.8 |
| CMNPD24393 | -7.8 |
| CMNPD21024 | -7.8 |
| CMNPD1763 | -7.8 |
| CMNPD10189 | -7.8 |
| CMNPD17540 | -7.8 |
| CMNPD24428 | -7.8 |
| CMNPD25879 | -7.8 |
| CMNPD30027 | -7.8 |
| CMNPD25735 | -7.8 |
| CMNPD15696 | -7.8 |
| CMNPD30187 | -7.8 |
| CMNPD23218 | -7.8 |
| CMNPD9392 | -7.8 |
| CMNPD14681 | -7.8 |
| CMNPD1762 | -7.8 |
| CMNPD4670 | -7.8 |
| CMNPD30061 | -7.8 |
| CMNPD28552 | -7.8 |
| CMNPD30039 | -7.8 |
| CMNPD7986 | -7.8 |
| CMNPD22322 | -7.8 |
| CMNPD19697 | -7.8 |
| CMNPD13889 | -7.8 |
| CMNPD26411 | -7.8 |
| CMNPD25808 | -7.8 |
| CMNPD24355 | -7.8 |
| CMNPD17535 | -7.8 |
| CMNPD20685 | -7.8 |
| CMNPD30024 | -7.8 |
| CMNPD29997 | -7.8 |
| CMNPD17584 | -7.8 |
| CMNPD28451 | -7.8 |
| CMNPD5305 | -7.8 |
| CMNPD6543 | -7.8 |
| CMNPD24391 | -7.8 |
| CMNPD17564 | -7.8 |
| CMNPD21889 | -7.8 |
| CMNPD24409 | -7.8 |
| CMNPD13211 | -7.8 |
| CMNPD28535 | -7.8 |
| CMNPD23254 | -7.8 |
| CMNPD28465 | -7.8 |
| CMNPD12468 | -7.7 |
| CMNPD20788 | -7.7 |
| CMNPD25790 | -7.7 |
| CMNPD28643 | -7.7 |
| CMNPD27176 | -7.7 |
| CMNPD3604 | -7.7 |
| CMNPD30011 | -7.7 |
| CMNPD23303 | -7.7 |
| CMNPD28611 | -7.7 |
| CMNPD21064 | -7.7 |
| CMNPD24440 | -7.7 |
| CMNPD15680 | -7.7 |
| CMNPD30080 | -7.7 |
| CMNPD25725 | -7.7 |
| CMNPD7996 | -7.7 |
| CMNPD25750 | -7.7 |
| CMNPD23248 | -7.7 |
| CMNPD23220 | -7.7 |
| CMNPD16570 | -7.7 |
| CMNPD27212 | -7.7 |
| CMNPD4127 | -7.7 |
| CMNPD16566 | -7.7 |
| CMNPD25739 | -7.7 |
| CMNPD23247 | -7.7 |
| CMNPD24967 | -7.7 |
| CMNPD24368 | -7.7 |
| CMNPD18868 | -7.7 |
| CMNPD27130 | -7.7 |
| CMNPD27118 | -7.7 |
| CMNPD21979 | -7.7 |
| CMNPD28518 | -7.7 |
| CMNPD24497 | -7.7 |
| CMNPD19648 | -7.7 |
| CMNPD23224 | -7.7 |
| CMNPD19647 | -7.7 |
| CMNPD30113 | -7.7 |
| CMNPD24363 | -7.7 |
| CMNPD19686 | -7.7 |
| CMNPD28658 | -7.7 |
| CMNPD30045 | -7.7 |
| CMNPD25791 | -7.7 |
| CMNPD30124 | -7.7 |
| CMNPD25789 | -7.7 |
| CMNPD28415 | -7.7 |
| CMNPD5310 | -7.7 |
| CMNPD30021 | -7.7 |
| CMNPD25785 | -7.7 |
| CMNPD14679 | -7.7 |
| CMNPD20741 | -7.7 |
| CMNPD26393 | -7.7 |
| CMNPD28412 | -7.7 |
| CMNPD25795 | -7.7 |
| CMNPD30183 | -7.7 |
| CMNPD30050 | -7.7 |
| CMNPD25786 | -7.7 |
| CMNPD16563 | -7.7 |
| CMNPD20743 | -7.7 |
| CMNPD11659 | -7.7 |
| CMNPD27227 | -7.7 |
| CMNPD24380 | -7.7 |
| CMNPD23150 | -7.7 |
| CMNPD21910 | -7.7 |
| CMNPD30176 | -7.7 |
| CMNPD19653 | -7.7 |
| CMNPD24425 | -7.7 |
| CMNPD30104 | -7.7 |
| CMNPD21896 | -7.7 |
| CMNPD26402 | -7.7 |
| CMNPD24517 | -7.7 |
| CMNPD23167 | -7.7 |
| CMNPD24467 | -7.7 |
| CMNPD27150 | -7.7 |
| CMNPD18612 | -7.7 |
| CMNPD21911 | -7.7 |
| CMNPD30043 | -7.7 |
| CMNPD4126 | -7.6 |
| CMNPD30064 | -7.6 |
| CMNPD30103 | -7.6 |
| CMNPD19692 | -7.6 |
| CMNPD25751 | -7.6 |
| CMNPD8748 | -7.6 |
| CMNPD17552 | -7.6 |
| CMNPD1765 | -7.6 |
| CMNPD17551 | -7.6 |
| CMNPD30040 | -7.6 |
| CMNPD13184 | -7.6 |
| CMNPD24514 | -7.6 |
| CMNPD15679 | -7.6 |
| CMNPD21977 | -7.6 |
| CMNPD28532 | -7.6 |
| CMNPD18662 | -7.6 |
| CMNPD19676 | -7.6 |
| CMNPD18610 | -7.6 |
| CMNPD28678 | -7.6 |
| CMNPD24452 | -7.6 |
| CMNPD28441 | -7.6 |
| CMNPD30041 | -7.6 |
| CMNPD11653 | -7.6 |
| CMNPD28604 | -7.6 |
| CMNPD24448 | -7.6 |
| CMNPD21978 | -7.6 |
| CMNPD21968 | -7.6 |
| CMNPD28416 | -7.6 |
| CMNPD19665 | -7.6 |
| CMNPD23255 | -7.6 |
| CMNPD24496 | -7.6 |
| CMNPD20742 | -7.6 |
| CMNPD28580 | -7.6 |
| CMNPD26394 | -7.6 |
| CMNPD24477 | -7.6 |
| CMNPD23227 | -7.6 |
| CMNPD30063 | -7.6 |
| CMNPD12471 | -7.6 |
| CMNPD14709 | -7.6 |
| CMNPD17541 | -7.6 |
| CMNPD24390 | -7.6 |
| CMNPD25730 | -7.6 |
| CMNPD30105 | -7.6 |
| CMNPD10896 | -7.6 |
| CMNPD15670 | -7.6 |
| CMNPD24469 | -7.6 |
| CMNPD25864 | -7.6 |
| CMNPD29941 | -7.6 |
| CMNPD13190 | -7.6 |
| CMNPD4130 | -7.6 |
| CMNPD30052 | -7.6 |
| CMNPD29999 | -7.6 |
| CMNPD12473 | -7.6 |
| CMNPD27186 | -7.6 |
| CMNPD28440 | -7.6 |
| CMNPD24450 | -7.6 |
| CMNPD20717 | -7.6 |
| CMNPD2189 | -7.6 |
| CMNPD13900 | -7.6 |
| CMNPD23297 | -7.6 |
| CMNPD20724 | -7.6 |
| CMNPD24470 | -7.6 |
| CMNPD30060 | -7.6 |
| CMNPD10982 | -7.6 |
| CMNPD14680 | -7.6 |
| CMNPD27189 | -7.6 |
| CMNPD1764 | -7.6 |
| CMNPD24434 | -7.6 |
| CMNPD24427 | -7.6 |
| CMNPD19925 | -7.6 |
| CMNPD20694 | -7.6 |
| CMNPD28595 | -7.5 |
| CMNPD13887 | -7.5 |
| CMNPD21934 | -7.5 |
| CMNPD17531 | -7.5 |
| CMNPD30055 | -7.5 |
| CMNPD30077 | -7.5 |
| CMNPD30173 | -7.5 |
| CMNPD21973 | -7.5 |
| CMNPD28657 | -7.5 |
| CMNPD6537 | -7.5 |
| CMNPD3605 | -7.5 |
| CMNPD20773 | -7.5 |
| CMNPD10895 | -7.5 |
| CMNPD10124 | -7.5 |
| CMNPD27282 | -7.5 |
| CMNPD4128 | -7.5 |
| CMNPD17546 | -7.5 |
| CMNPD24501 | -7.5 |
| CMNPD29995 | -7.5 |
| CMNPD25782 | -7.5 |
| CMNPD13214 | -7.5 |
| CMNPD30059 | -7.5 |
| CMNPD28607 | -7.5 |
| CMNPD28431 | -7.5 |
| CMNPD24449 | -7.5 |
| CMNPD18609 | -7.5 |
| CMNPD11664 | -7.5 |
| CMNPD16552 | -7.5 |
| CMNPD24463 | -7.5 |
| CMNPD28466 | -7.5 |
| CMNPD25746 | -7.5 |
| CMNPD25881 | -7.5 |
| CMNPD23301 | -7.5 |
| CMNPD24394 | -7.5 |
| CMNPD24432 | -7.5 |
| CMNPD26391 | -7.5 |
| CMNPD21947 | -7.5 |
| CMNPD19691 | -7.5 |
| CMNPD24516 | -7.5 |
| CMNPD19716 | -7.5 |
| CMNPD17583 | -7.5 |
| CMNPD19690 | -7.5 |
| CMNPD24353 | -7.5 |
| CMNPD28675 | -7.5 |
| CMNPD21935 | -7.5 |
| CMNPD25865 | -7.5 |
| CMNPD22320 | -7.5 |
| CMNPD14695 | -7.5 |
| CMNPD22283 | -7.5 |
| CMNPD22284 | -7.5 |
| CMNPD15803 | -7.5 |
| CMNPD19726 | -7.5 |
| CMNPD7281 | -7.5 |
| CMNPD30042 | -7.5 |
| CMNPD21890 | -7.5 |
| CMNPD19712 | -7.5 |
| CMNPD27194 | -7.5 |
| CMNPD20762 | -7.5 |
| CMNPD24435 | -7.5 |
| CMNPD17547 | -7.5 |
| CMNPD8788 | -7.5 |
| CMNPD19663 | -7.5 |
| CMNPD20693 | -7.5 |
| CMNPD21967 | -7.5 |
| CMNPD30033 | -7.5 |
| CMNPD30028 | -7.5 |
| CMNPD24431 | -7.5 |
| CMNPD23148 | -7.5 |
| CMNPD25804 | -7.5 |
| CMNPD23219 | -7.5 |
| CMNPD28515 | -7.5 |
| CMNPD19683 | -7.5 |
| CMNPD21964 | -7.5 |
| CMNPD18679 | -7.5 |
| CMNPD5335 | -7.5 |
| CMNPD14682 | -7.5 |
| CMNPD28409 | -7.5 |
| CMNPD10985 | -7.5 |
| CMNPD24328 | -7.5 |
| CMNPD25738 | -7.5 |
| CMNPD30065 | -7.5 |
| CMNPD13259 | -7.5 |
| CMNPD28548 | -7.5 |
| CMNPD13189 | -7.5 |
| CMNPD10889 | -7.5 |
| CMNPD17548 | -7.5 |
| CMNPD25776 | -7.4 |
| CMNPD24403 | -7.4 |
| CMNPD5301 | -7.4 |
| CMNPD24346 | -7.4 |
| CMNPD30004 | -7.4 |
| CMNPD16703 | -7.4 |
| CMNPD18866 | -7.4 |
| CMNPD13264 | -7.4 |
| CMNPD12479 | -7.4 |
| CMNPD23253 | -7.4 |
| CMNPD8749 | -7.4 |
| CMNPD21904 | -7.4 |
| CMNPD30014 | -7.4 |
| CMNPD27158 | -7.4 |
| CMNPD28476 | -7.4 |
| CMNPD23206 | -7.4 |
| CMNPD14683 | -7.4 |
| CMNPD20677 | -7.4 |
| CMNPD25747 | -7.4 |
| CMNPD12485 | -7.4 |
| CMNPD24388 | -7.4 |
| CMNPD20737 | -7.4 |
| CMNPD17565 | -7.4 |
| CMNPD30114 | -7.4 |
| CMNPD19711 | -7.4 |
| CMNPD19656 | -7.4 |
| CMNPD27112 | -7.4 |
| CMNPD30009 | -7.4 |
| CMNPD21985 | -7.4 |
| CMNPD21959 | -7.4 |
| CMNPD20699 | -7.4 |
| CMNPD27126 | -7.4 |
| CMNPD6534 | -7.4 |
| CMNPD17580 | -7.4 |
| CMNPD29991 | -7.4 |
| CMNPD29932 | -7.4 |
| CMNPD12463 | -7.4 |
| CMNPD24429 | -7.4 |
| CMNPD17536 | -7.4 |
| CMNPD25855 | -7.4 |
| CMNPD4669 | -7.4 |
| CMNPD27231 | -7.4 |
| CMNPD16568 | -7.4 |
| CMNPD23214 | -7.4 |
| CMNPD24442 | -7.4 |
| CMNPD25740 | -7.4 |
| CMNPD24479 | -7.4 |
| CMNPD23302 | -7.4 |
| CMNPD28581 | -7.4 |
| CMNPD25742 | -7.4 |
| CMNPD24352 | -7.4 |
| CMNPD21051 | -7.4 |
| CMNPD21945 | -7.4 |
| CMNPD28502 | -7.4 |
| CMNPD20740 | -7.4 |
| CMNPD24381 | -7.4 |
| CMNPD28659 | -7.4 |
| CMNPD25875 | -7.4 |
| CMNPD18659 | -7.4 |
| CMNPD30038 | -7.4 |
| CMNPD28442 | -7.4 |
| CMNPD27178 | -7.4 |
| CMNPD25793 | -7.4 |
| CMNPD30025 | -7.4 |
| CMNPD12483 | -7.4 |
| CMNPD3603 | -7.4 |
| CMNPD22290 | -7.4 |
| CMNPD21928 | -7.4 |
| CMNPD13280 | -7.4 |
| CMNPD25884 | -7.4 |
| CMNPD20679 | -7.4 |
| CMNPD9390 | -7.4 |
| CMNPD25778 | -7.4 |
| CMNPD21963 | -7.4 |
| CMNPD30091 | -7.4 |
| CMNPD19955 | -7.4 |
| CMNPD23204 | -7.4 |
| CMNPD19723 | -7.4 |
| CMNPD25805 | -7.4 |
| CMNPD20739 | -7.4 |
| CMNPD21943 | -7.4 |
| CMNPD20747 | -7.4 |
| CMNPD14777 | -7.4 |
| CMNPD17545 | -7.4 |
| CMNPD27280 | -7.4 |
| CMNPD30026 | -7.4 |
| CMNPD22319 | -7.4 |
| CMNPD24499 | -7.4 |
| CMNPD15677 | -7.4 |
| CMNPD30186 | -7.4 |
| CMNPD16565 | -7.4 |
| CMNPD25748 | -7.3 |
| CMNPD15694 | -7.3 |
| CMNPD25812 | -7.3 |
| CMNPD10117 | -7.3 |
| CMNPD15781 | -7.3 |
| CMNPD24507 | -7.3 |
| CMNPD20778 | -7.3 |
| CMNPD15775 | -7.3 |
| CMNPD25883 | -7.3 |
| CMNPD4520 | -7.3 |
| CMNPD25736 | -7.3 |
| CMNPD25882 | -7.3 |
| CMNPD29935 | -7.3 |
| CMNPD30000 | -7.3 |
| CMNPD14685 | -7.3 |
| CMNPD18613 | -7.3 |
| CMNPD25777 | -7.3 |
| CMNPD27127 | -7.3 |
| CMNPD21893 | -7.3 |
| CMNPD14693 | -7.3 |
| CMNPD23156 | -7.3 |
| CMNPD19737 | -7.3 |
| CMNPD19694 | -7.3 |
| CMNPD28432 | -7.3 |
| CMNPD24509 | -7.3 |
| CMNPD18665 | -7.3 |
| CMNPD28486 | -7.3 |
| CMNPD30022 | -7.3 |
| CMNPD15774 | -7.3 |
| CMNPD17543 | -7.3 |
| CMNPD27187 | -7.3 |
| CMNPD27185 | -7.3 |
| CMNPD27278 | -7.3 |
| CMNPD30066 | -7.3 |
| CMNPD27204 | -7.3 |
| CMNPD30200 | -7.3 |
| CMNPD25753 | -7.3 |
| CMNPD23288 | -7.3 |
| CMNPD29929 | -7.3 |
| CMNPD16554 | -7.3 |
| CMNPD24399 | -7.3 |
| CMNPD16713 | -7.3 |
| CMNPD24454 | -7.3 |
| CMNPD28586 | -7.3 |
| CMNPD20680 | -7.3 |
| CMNPD25792 | -7.3 |
| CMNPD24354 | -7.3 |
| CMNPD20755 | -7.3 |
| CMNPD14706 | -7.3 |
| CMNPD24423 | -7.3 |
| CMNPD27281 | -7.3 |
| CMNPD17577 | -7.3 |
| CMNPD30056 | -7.3 |
| CMNPD23151 | -7.3 |
| CMNPD20723 | -7.3 |
| CMNPD21067 | -7.3 |
| CMNPD27242 | -7.3 |
| CMNPD20692 | -7.3 |
| CMNPD30170 | -7.3 |
| CMNPD19654 | -7.3 |
| CMNPD16551 | -7.3 |
| CMNPD13901 | -7.3 |
| CMNPD27782 | -7.3 |
| CMNPD20779 | -7.3 |
| CMNPD18673 | -7.3 |
| CMNPD29934 | -7.3 |
| CMNPD24504 | -7.3 |
| CMNPD10983 | -7.3 |
| CMNPD11660 | -7.3 |
| CMNPD21927 | -7.3 |
| CMNPD28433 | -7.3 |
| CMNPD18624 | -7.3 |
| CMNPD28687 | -7.3 |
| CMNPD23166 | -7.3 |
| CMNPD11663 | -7.3 |
| CMNPD24456 | -7.2 |
| CMNPD30015 | -7.2 |
| CMNPD27230 | -7.2 |
| CMNPD19954 | -7.2 |
| CMNPD27122 | -7.2 |
| CMNPD23232 | -7.2 |
| CMNPD24498 | -7.2 |
| CMNPD25767 | -7.2 |
| CMNPD8750 | -7.2 |
| CMNPD28540 | -7.2 |
| CMNPD30051 | -7.2 |
| CMNPD20716 | -7.2 |
| CMNPD30093 | -7.2 |
| CMNPD28641 | -7.2 |
| CMNPD25724 | -7.2 |
| CMNPD27208 | -7.2 |
| CMNPD24487 | -7.2 |
| CMNPD23244 | -7.2 |
| CMNPD27128 | -7.2 |
| CMNPD30238 | -7.2 |
| CMNPD27139 | -7.2 |
| CMNPD17568 | -7.2 |
| CMNPD23611 | -7.2 |
| CMNPD15672 | -7.2 |
| CMNPD17555 | -7.2 |
| CMNPD10903 | -7.2 |
| CMNPD24970 | -7.2 |
| CMNPD28477 | -7.2 |
| CMNPD22308 | -7.2 |
| CMNPD18872 | -7.2 |
| CMNPD19901 | -7.2 |
| CMNPD23229 | -7.2 |
| CMNPD9461 | -7.2 |
| CMNPD12393 | -7.2 |
| CMNPD15678 | -7.2 |
| CMNPD11708 | -7.2 |
| CMNPD8751 | -7.2 |
| CMNPD15700 | -7.2 |
| CMNPD19714 | -7.2 |
| CMNPD29936 | -7.2 |
| CMNPD28474 | -7.2 |
| CMNPD18667 | -7.2 |
| CMNPD22291 | -7.2 |
| CMNPD11658 | -7.2 |
| CMNPD17539 | -7.2 |
| CMNPD25731 | -7.2 |
| CMNPD21054 | -7.2 |
| CMNPD30071 | -7.2 |
| CMNPD28649 | -7.2 |
| CMNPD6541 | -7.2 |
| CMNPD17728 | -7.2 |
| CMNPD13193 | -7.2 |
| CMNPD23205 | -7.2 |
| CMNPD19710 | -7.2 |
| CMNPD27211 | -7.2 |
| CMNPD30079 | -7.2 |
| CMNPD28480 | -7.2 |
| CMNPD30167 | -7.2 |
| CMNPD21938 | -7.2 |
| CMNPD23296 | -7.2 |
| CMNPD28647 | -7.2 |
| CMNPD27262 | -7.2 |
| CMNPD19908 | -7.2 |
| CMNPD17528 | -7.2 |
| CMNPD19693 | -7.2 |
| CMNPD16708 | -7.2 |
| CMNPD20868 | -7.2 |
| CMNPD28679 | -7.2 |
| CMNPD17571 | -7.2 |
| CMNPD10125 | -7.2 |
| CMNPD19689 | -7.2 |
| CMNPD13893 | -7.2 |
| CMNPD27124 | -7.2 |
| CMNPD24466 | -7.2 |
| CMNPD28545 | -7.2 |
| CMNPD30023 | -7.2 |
| CMNPD28585 | -7.2 |
| CMNPD18563 | -7.2 |
| CMNPD18677 | -7.2 |
| CMNPD30002 | -7.2 |
| CMNPD24436 | -7.2 |
| CMNPD25829 | -7.2 |
| CMNPD21984 | -7.2 |
| CMNPD331 | -7.2 |
| CMNPD21029 | -7.2 |
| CMNPD24976 | -7.2 |
| CMNPD21976 | -7.2 |
| CMNPD13188 | -7.2 |
| CMNPD27182 | -7.2 |
| CMNPD30069 | -7.2 |
| CMNPD7283 | -7.2 |
| CMNPD27237 | -7.2 |
| CMNPD10904 | -7.1 |
| CMNPD16705 | -7.1 |
| CMNPD24439 | -7.1 |
| CMNPD12465 | -7.1 |
| CMNPD20676 | -7.1 |
| CMNPD20751 | -7.1 |
| CMNPD21913 | -7.1 |
| CMNPD11650 | -7.1 |
| CMNPD24349 | -7.1 |
| CMNPD28534 | -7.1 |
| CMNPD332 | -7.1 |
| CMNPD2190 | -7.1 |
| CMNPD4125 | -7.1 |
| CMNPD30177 | -7.1 |
| CMNPD24441 | -7.1 |
| CMNPD28472 | -7.1 |
| CMNPD30075 | -7.1 |
| CMNPD1746 | -7.1 |
| CMNPD15671 | -7.1 |
| CMNPD28473 | -7.1 |
| CMNPD1760 | -7.1 |
| CMNPD20769 | -7.1 |
| CMNPD2571 | -7.1 |
| CMNPD25885 | -7.1 |
| CMNPD14775 | -7.1 |
| CMNPD29931 | -7.1 |
| CMNPD27177 | -7.1 |
| CMNPD18623 | -7.1 |
| CMNPD24373 | -7.1 |
| CMNPD333 | -7.1 |
| CMNPD22293 | -7.1 |
| CMNPD15692 | -7.1 |
| CMNPD24494 | -7.1 |
| CMNPD23266 | -7.1 |
| CMNPD25769 | -7.1 |
| CMNPD23287 | -7.1 |
| CMNPD30214 | -7.1 |
| CMNPD16735 | -7.1 |
| CMNPD17542 | -7.1 |
| CMNPD28483 | -7.1 |
| CMNPD24460 | -7.1 |
| CMNPD24362 | -7.1 |
| CMNPD29992 | -7.1 |
| CMNPD19688 | -7.1 |
| CMNPD310 | -7.1 |
| CMNPD27129 | -7.1 |
| CMNPD28662 | -7.1 |
| CMNPD25809 | -7.1 |
| CMNPD27777 | -7.1 |
| CMNPD3612 | -7.1 |
| CMNPD14689 | -7.1 |
| CMNPD18661 | -7.1 |
| CMNPD24364 | -7.1 |
| CMNPD10980 | -7.1 |
| CMNPD19914 | -7.1 |
| CMNPD27768 | -7.1 |
| CMNPD28591 | -7.1 |
| CMNPD17727 | -7.1 |
| CMNPD25834 | -7.1 |
| CMNPD18625 | -7.1 |
| CMNPD20675 | -7.1 |
| CMNPD17582 | -7.1 |
| CMNPD24971 | -7.1 |
| CMNPD20726 | -7.1 |
| CMNPD21936 | -7.1 |
| CMNPD24344 | -7.1 |
| CMNPD29948 | -7.1 |
| CMNPD22288 | -7.1 |
| CMNPD18884 | -7.1 |
| CMNPD9460 | -7.1 |
| CMNPD23614 | -7.1 |
| CMNPD28563 | -7.1 |
| CMNPD28543 | -7.1 |
| CMNPD15698 | -7.1 |
| CMNPD29996 | -7.1 |
| CMNPD28514 | -7.1 |
| CMNPD27125 | -7.1 |
| CMNPD30147 | -7.1 |
| CMNPD27253 | -7.1 |
| CMNPD24379 | -7.1 |
| CMNPD25732 | -7.1 |
| CMNPD30092 | -7.1 |
| CMNPD27277 | -7.1 |
| CMNPD28485 | -7.1 |
| CMNPD25819 | -7.1 |
| CMNPD29993 | -7.1 |
| CMNPD19738 | -7.1 |
| CMNPD28635 | -7.1 |
| CMNPD21954 | -7.1 |
| CMNPD17572 | -7.1 |
| CMNPD28481 | -7.1 |
| CMNPD8752 | -7.1 |
| CMNPD18635 | -7.1 |
| CMNPD24482 | -7 |
| CMNPD23193 | -7 |
| CMNPD27107 | -7 |
| CMNPD24864 | -7 |
| CMNPD23268 | -7 |
| CMNPD28560 | -7 |
| CMNPD28417 | -7 |
| CMNPD27192 | -7 |
| CMNPD28588 | -7 |
| CMNPD25772 | -7 |
| CMNPD20710 | -7 |
| CMNPD24370 | -7 |
| CMNPD19669 | -7 |
| CMNPD18648 | -7 |
| CMNPD18634 | -7 |
| CMNPD24396 | -7 |
| CMNPD7987 | -7 |
| CMNPD15705 | -7 |
| CMNPD24392 | -7 |
| CMNPD25766 | -7 |
| CMNPD15682 | -7 |
| CMNPD24462 | -7 |
| CMNPD30221 | -7 |
| CMNPD24478 | -7 |
| CMNPD27174 | -7 |
| CMNPD12478 | -7 |
| CMNPD27193 | -7 |
| CMNPD27175 | -7 |
| CMNPD9462 | -7 |
| CMNPD28551 | -7 |
| CMNPD13257 | -7 |
| CMNPD18660 | -7 |
| CMNPD11652 | -7 |
| CMNPD28673 | -7 |
| CMNPD23208 | -7 |
| CMNPD3606 | -7 |
| CMNPD10972 | -7 |
| CMNPD26401 | -7 |
| CMNPD26400 | -7 |
| CMNPD18607 | -7 |
| CMNPD18669 | -7 |
| CMNPD22292 | -7 |
| CMNPD25886 | -7 |
| CMNPD30100 | -7 |
| CMNPD27123 | -7 |
| CMNPD21974 | -7 |
| CMNPD22281 | -7 |
| CMNPD20756 | -7 |
| CMNPD27191 | -7 |
| CMNPD27105 | -7 |
| CMNPD23221 | -7 |
| CMNPD21031 | -7 |
| CMNPD22296 | -7 |
| CMNPD17533 | -7 |
| CMNPD26395 | -7 |
| CMNPD24372 | -7 |
| CMNPD28606 | -7 |
| CMNPD8737 | -7 |
| CMNPD14696 | -7 |
| CMNPD28661 | -7 |
| CMNPD23260 | -7 |
| CMNPD23149 | -7 |
| CMNPD25813 | -7 |
| CMNPD23185 | -7 |
| CMNPD19721 | -7 |
| CMNPD22287 | -7 |
| CMNPD30106 | -7 |
| CMNPD20754 | -7 |
| CMNPD25811 | -7 |
| CMNPD25868 | -7 |
| CMNPD27232 | -7 |
| CMNPD19718 | -7 |
| CMNPD20709 | -7 |
| CMNPD23300 | -7 |
| CMNPD21888 | -7 |
| CMNPD28677 | -7 |
| CMNPD18626 | -7 |
| CMNPD10890 | -7 |
| CMNPD23278 | -7 |
| CMNPD28608 | -7 |
| CMNPD28529 | -7 |
| CMNPD17575 | -7 |
| CMNPD7275 | -7 |
| CMNPD24865 | -7 |
| CMNPD11716 | -7 |
| CMNPD10111 | -7 |
| CMNPD14780 | -7 |
| CMNPD8019 | -7 |
| CMNPD17538 | -7 |
| CMNPD18666 | -7 |
| CMNPD23160 | -7 |
| CMNPD18882 | -7 |
| CMNPD23233 | -7 |
| CMNPD19700 | -7 |
| CMNPD10964 | -6.9 |
| CMNPD30199 | -6.9 |
| CMNPD11655 | -6.9 |
| CMNPD13213 | -6.9 |
| CMNPD24405 | -6.9 |
| CMNPD24438 | -6.9 |
| CMNPD28664 | -6.9 |
| CMNPD25870 | -6.9 |
| CMNPD9403 | -6.9 |
| CMNPD24421 | -6.9 |
| CMNPD27773 | -6.9 |
| CMNPD24419 | -6.9 |
| CMNPD11709 | -6.9 |
| CMNPD24975 | -6.9 |
| CMNPD13212 | -6.9 |
| CMNPD30146 | -6.9 |
| CMNPD23264 | -6.9 |
| CMNPD8021 | -6.9 |
| CMNPD27209 | -6.9 |
| CMNPD26421 | -6.9 |
| CMNPD5302 | -6.9 |
| CMNPD23154 | -6.9 |
| CMNPD27117 | -6.9 |
| CMNPD28500 | -6.9 |
| CMNPD20745 | -6.9 |
| CMNPD16547 | -6.9 |
| CMNPD1024 | -6.9 |
| CMNPD30171 | -6.9 |
| CMNPD30102 | -6.9 |
| CMNPD30175 | -6.9 |
| CMNPD30210 | -6.9 |
| CMNPD25810 | -6.9 |
| CMNPD23299 | -6.9 |
| CMNPD30218 | -6.9 |
| CMNPD238 | -6.9 |
| CMNPD11651 | -6.9 |
| CMNPD18658 | -6.9 |
| CMNPD27217 | -6.9 |
| CMNPD25889 | -6.9 |
| CMNPD27133 | -6.9 |
| CMNPD19907 | -6.9 |
| CMNPD30232 | -6.9 |
| CMNPD28471 | -6.9 |
| CMNPD28541 | -6.9 |
| CMNPD13197 | -6.9 |
| CMNPD3147 | -6.9 |
| CMNPD13281 | -6.9 |
| CMNPD13302 | -6.9 |
| CMNPD27155 | -6.9 |
| CMNPD17587 | -6.9 |
| CMNPD12469 | -6.9 |
| CMNPD28475 | -6.9 |
| CMNPD27119 | -6.9 |
| CMNPD14778 | -6.9 |
| CMNPD18650 | -6.9 |
| CMNPD30165 | -6.9 |
| CMNPD13888 | -6.9 |
| CMNPD23236 | -6.9 |
| CMNPD28503 | -6.9 |
| CMNPD30206 | -6.9 |
| CMNPD15690 | -6.9 |
| CMNPD24387 | -6.9 |
| CMNPD28693 | -6.9 |
| CMNPD16553 | -6.9 |
| CMNPD326 | -6.9 |
| CMNPD27770 | -6.9 |
| CMNPD8787 | -6.9 |
| CMNPD17604 | -6.9 |
| CMNPD30174 | -6.9 |
| CMNPD17567 | -6.9 |
| CMNPD19715 | -6.9 |
| CMNPD27183 | -6.9 |
| CMNPD20673 | -6.9 |
| CMNPD11711 | -6.9 |
| CMNPD24422 | -6.9 |
| CMNPD27184 | -6.9 |
| CMNPD28530 | -6.9 |
| CMNPD24961 | -6.9 |
| CMNPD26423 | -6.9 |
| CMNPD28482 | -6.9 |
| CMNPD14697 | -6.9 |
| CMNPD23240 | -6.9 |
| CMNPD18904 | -6.9 |
| CMNPD30194 | -6.9 |
| CMNPD19698 | -6.9 |
| CMNPD30159 | -6.9 |
| CMNPD14699 | -6.9 |
| CMNPD19911 | -6.9 |
| CMNPD29947 | -6.9 |
| CMNPD20736 | -6.9 |
| CMNPD15693 | -6.9 |
| CMNPD23176 | -6.9 |
| CMNPD4129 | -6.9 |
| CMNPD28561 | -6.9 |
| CMNPD13261 | -6.9 |
| CMNPD29942 | -6.9 |
| CMNPD5857 | -6.9 |
| CMNPD14690 | -6.9 |
| CMNPD20725 | -6.9 |
| CMNPD20727 | -6.9 |
| CMNPD24371 | -6.9 |
| CMNPD13272 | -6.9 |
| CMNPD10178 | -6.9 |
| CMNPD24455 | -6.9 |
| CMNPD28663 | -6.9 |
| CMNPD17718 | -6.9 |
| CMNPD15683 | -6.9 |
| CMNPD25820 | -6.9 |
| CMNPD4138 | -6.9 |
| CMNPD10180 | -6.9 |
| CMNPD24365 | -6.8 |
| CMNPD23184 | -6.8 |
| CMNPD28533 | -6.8 |
| CMNPD22294 | -6.8 |
| CMNPD27779 | -6.8 |
| CMNPD12419 | -6.8 |
| CMNPD12488 | -6.8 |
| CMNPD30099 | -6.8 |
| CMNPD20749 | -6.8 |
| CMNPD18649 | -6.8 |
| CMNPD17559 | -6.8 |
| CMNPD15684 | -6.8 |
| CMNPD30158 | -6.8 |
| CMNPD17534 | -6.8 |
| CMNPD23231 | -6.8 |
| CMNPD27116 | -6.8 |
| CMNPD21937 | -6.8 |
| CMNPD28479 | -6.8 |
| CMNPD19902 | -6.8 |
| CMNPD16707 | -6.8 |
| CMNPD23261 | -6.8 |
| CMNPD18687 | -6.8 |
| CMNPD3610 | -6.8 |
| CMNPD30182 | -6.8 |
| CMNPD20700 | -6.8 |
| CMNPD27236 | -6.8 |
| CMNPD24958 | -6.8 |
| CMNPD30178 | -6.8 |
| CMNPD24343 | -6.8 |
| CMNPD28516 | -6.8 |
| CMNPD17741 | -6.8 |
| CMNPD23269 | -6.8 |
| CMNPD28636 | -6.8 |
| CMNPD24505 | -6.8 |
| CMNPD24407 | -6.8 |
| CMNPD21983 | -6.8 |
| CMNPD7988 | -6.8 |
| CMNPD17527 | -6.8 |
| CMNPD21949 | -6.8 |
| CMNPD27190 | -6.8 |
| CMNPD17526 | -6.8 |
| CMNPD15800 | -6.8 |
| CMNPD1745 | -6.8 |
| CMNPD28562 | -6.8 |
| CMNPD29955 | -6.8 |
| CMNPD13182 | -6.8 |
| CMNPD23182 | -6.8 |
| CMNPD8742 | -6.8 |
| CMNPD23241 | -6.8 |
| CMNPD23152 | -6.8 |
| CMNPD23293 | -6.8 |
| CMNPD18651 | -6.8 |
| CMNPD12470 | -6.8 |
| CMNPD28634 | -6.8 |
| CMNPD12462 | -6.8 |
| CMNPD30204 | -6.8 |
| CMNPD18656 | -6.8 |
| CMNPD19717 | -6.8 |
| CMNPD17581 | -6.8 |
| CMNPD27778 | -6.8 |
| CMNPD29980 | -6.8 |
| CMNPD8757 | -6.8 |
| CMNPD29950 | -6.8 |
| CMNPD17586 | -6.8 |
| CMNPD27115 | -6.8 |
| CMNPD28526 | -6.8 |
| CMNPD16546 | -6.8 |
| CMNPD19708 | -6.8 |
| CMNPD4648 | -6.8 |
| CMNPD2197 | -6.8 |
| CMNPD18636 | -6.8 |
| CMNPD12474 | -6.8 |
| CMNPD28690 | -6.8 |
| CMNPD24488 | -6.8 |
| CMNPD10121 | -6.8 |
| CMNPD28579 | -6.8 |
| CMNPD13902 | -6.8 |
| CMNPD20681 | -6.8 |
| CMNPD27109 | -6.8 |
| CMNPD27213 | -6.8 |
| CMNPD23159 | -6.8 |
| CMNPD23251 | -6.8 |
| CMNPD27207 | -6.8 |
| CMNPD18688 | -6.8 |
| CMNPD342 | -6.8 |
| CMNPD13301 | -6.8 |
| CMNPD28567 | -6.8 |
| CMNPD339 | -6.8 |
| CMNPD10181 | -6.8 |
| CMNPD23243 | -6.8 |
| CMNPD27234 | -6.8 |
| CMNPD23230 | -6.8 |
| CMNPD17724 | -6.8 |
| CMNPD28546 | -6.8 |
| CMNPD17560 | -6.8 |
| CMNPD21884 | -6.8 |
| CMNPD27171 | -6.8 |
| CMNPD22282 | -6.8 |
| CMNPD25773 | -6.8 |
| CMNPD27131 | -6.8 |
| CMNPD23258 | -6.8 |
| CMNPD17588 | -6.8 |
| CMNPD23270 | -6.8 |
| CMNPD27781 | -6.8 |
| CMNPD24495 | -6.8 |
| CMNPD28484 | -6.8 |
| CMNPD18657 | -6.8 |
| CMNPD30152 | -6.8 |
| CMNPD20734 | -6.8 |
| CMNPD7247 | -6.8 |
| CMNPD10963 | -6.8 |
| CMNPD30208 | -6.8 |
| CMNPD28632 | -6.8 |
| CMNPD28640 | -6.8 |
| CMNPD17574 | -6.7 |
| CMNPD28627 | -6.7 |
| CMNPD27240 | -6.7 |
| CMNPD27223 | -6.7 |
| CMNPD16732 | -6.7 |
| CMNPD30163 | -6.7 |
| CMNPD20735 | -6.7 |
| CMNPD21953 | -6.7 |
| CMNPD19918 | -6.7 |
| CMNPD22286 | -6.7 |
| CMNPD10119 | -6.7 |
| CMNPD5856 | -6.7 |
| CMNPD10175 | -6.7 |
| CMNPD28602 | -6.7 |
| CMNPD3611 | -6.7 |
| CMNPD23286 | -6.7 |
| CMNPD13968 | -6.7 |
| CMNPD10191 | -6.7 |
| CMNPD24446 | -6.7 |
| CMNPD15674 | -6.7 |
| CMNPD30184 | -6.7 |
| CMNPD30185 | -6.7 |
| CMNPD21946 | -6.7 |
| CMNPD18608 | -6.7 |
| CMNPD12380 | -6.7 |
| CMNPD24503 | -6.7 |
| CMNPD19905 | -6.7 |
| CMNPD28599 | -6.7 |
| CMNPD20674 | -6.7 |
| CMNPD15779 | -6.7 |
| CMNPD12466 | -6.7 |
| CMNPD23276 | -6.7 |
| CMNPD16575 | -6.7 |
| CMNPD15770 | -6.7 |
| CMNPD19909 | -6.7 |
| CMNPD23613 | -6.7 |
| CMNPD17573 | -6.7 |
| CMNPD28542 | -6.7 |
| CMNPD24513 | -6.7 |
| CMNPD28547 | -6.7 |
| CMNPD14688 | -6.7 |
| CMNPD12481 | -6.7 |
| CMNPD27181 | -6.7 |
| CMNPD25728 | -6.7 |
| CMNPD21887 | -6.7 |
| CMNPD30193 | -6.7 |
| CMNPD28672 | -6.7 |
| CMNPD21940 | -6.7 |
| CMNPD30155 | -6.7 |
| CMNPD13203 | -6.7 |
| CMNPD21915 | -6.7 |
| CMNPD17731 | -6.7 |
| CMNPD28435 | -6.7 |
| CMNPD19900 | -6.7 |
| CMNPD20761 | -6.7 |
| CMNPD27224 | -6.7 |
| CMNPD23282 | -6.7 |
| CMNPD7271 | -6.7 |
| CMNPD8020 | -6.7 |
| CMNPD18655 | -6.7 |
| CMNPD21939 | -6.7 |
| CMNPD27202 | -6.7 |
| CMNPD14694 | -6.7 |
| CMNPD7233 | -6.7 |
| CMNPD25850 | -6.7 |
| CMNPD30172 | -6.7 |
| CMNPD9401 | -6.7 |
| CMNPD9463 | -6.7 |
| CMNPD23178 | -6.7 |
| CMNPD21877 | -6.7 |
| CMNPD28573 | -6.7 |
| CMNPD239 | -6.7 |
| CMNPD30164 | -6.7 |
| CMNPD28505 | -6.7 |
| CMNPD6535 | -6.7 |
| CMNPD19913 | -6.7 |
| CMNPD30087 | -6.7 |
| CMNPD23143 | -6.7 |
| CMNPD20730 | -6.7 |
| CMNPD30084 | -6.7 |
| CMNPD7230 | -6.7 |
| CMNPD27233 | -6.7 |
| CMNPD23242 | -6.7 |
| CMNPD20733 | -6.7 |
| CMNPD20682 | -6.7 |
| CMNPD27162 | -6.7 |
| CMNPD15778 | -6.7 |
| CMNPD20760 | -6.7 |
| CMNPD28461 | -6.7 |
| CMNPD20708 | -6.7 |
| CMNPD15681 | -6.7 |
| CMNPD8755 | -6.7 |
| CMNPD24385 | -6.7 |
| CMNPD13198 | -6.7 |

**S2 Table** - Compounds and its compound ID with the smiles.

| S. No. | Compound IDs | SMILES |
| --- | --- | --- |
| 1 | CMNPD25830 | c1c([C@](c2ccc(c(C[C@@H]3NC(=O)[C@]([H])(CCC4)N4C3=O)c[nH]5)c5c2)(C[C@@]([H])(C(=O)N(CCC6)[C@]6([H])C7=O)N78)[C@@]8([H])N9)c9ccc1 |
| 2 | CMNPD18675 | C1[C@@]2([C@](Nc(ccc3)c2c3)([H])N(C([C@@H](C)NC4=O)=O)[C@]14[H])c5ccc(c(C[C@@H]6C(=O)N(CCC7)[C@@]7([H])C(=O)N6)c[nH]8)c8c5 |
| 3 | CMNPD18676 | N12[C@@H](C(=O)N3[C@](C1=O)([H])C[C@@]4([C@]3(Nc(ccc5)c4c5)[H])c6ccc(c(C[C@@H]7C(=O)N(CCC8)[C@@]8([H])C(=O)N7)c[nH]9)c9c6)CCC2 |
| 4 | CMNPD27216 | c1cc(N[C@@H](N(C(=O)[C@H](CCC2)N2C3=O)[C@@H]3C4)[C@@]45c6ccc([nH]cc7C[C@@H]8NC(=O)[C@@H](CCC9)N9C8=O)c7c6)c5cc1 |


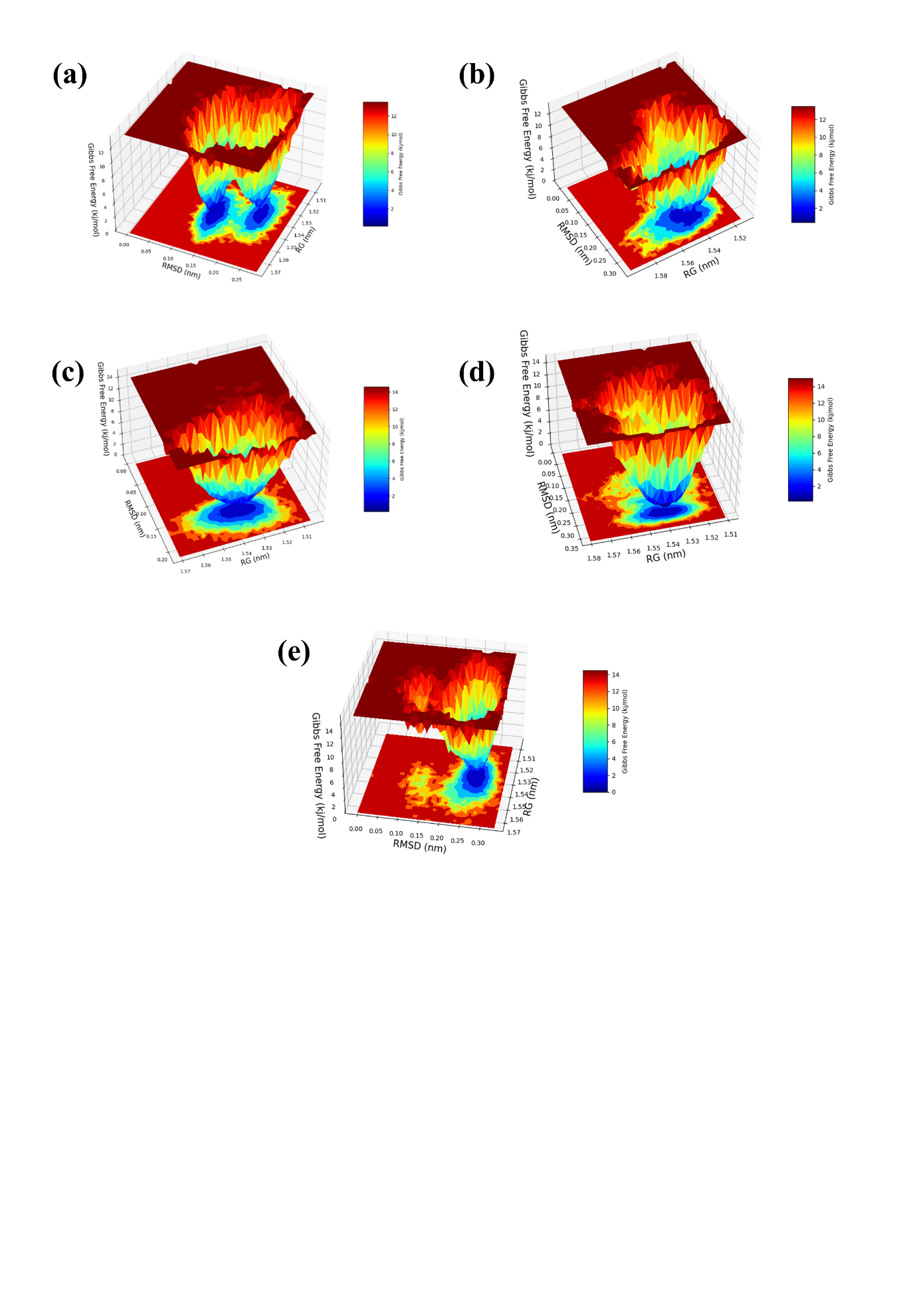


**S1 Fig-** 3D plots of free energy landscape of the selected complexes with the target influenza A H5N1 and compounds i.e., (a) CMNPD25830 (b) CMNPD18675 (c) CMNPD18676 (d) CMNPD27216 and (e) Control.
